# Performance analysis of MIMO-assisted HPMA networks over keyhole effect

**DOI:** 10.1371/journal.pone.0361616

**Published:** 2026-11-02

**Authors:** Chi-Bao Le, Anh-Tu Le, Phu Tran Tin, Sang-Quang Nguyen, Miroslav Voznak

**Affiliations:** 1 Faculty of Information Technology, Ton Duc Thang University, Ho Chi Minh City, Vietnam; 2 Faculty of Electrical Engineering and Computer Science, VSB-Technical University of Ostrava, Ostrava, Czechia; 3 Wireless Communication Laboratory, Posts and Telecommunications Institute of Technology, Ho Chi Minh City, Vietnam; Universite Cote d'Azur, FRANCE

## Abstract

As an innovative multiple access technology, hybrid power-frequency multiple access (HPMA) effectively supports massive connectivity while eliminating the decoding complexity associated with successive interference cancellation (SIC). Concurrently, although the keyhole effect represents a critical propagation degradation that drastically reduces diversity gains in wireless channels, its impact on advanced multiple-access techniques remains largely explored. Driven by these considerations, this paper investigates a comprehensive performance evaluation of a multiple-input multiple-output (MIMO)-assisted HPMA framework deployed over keyhole Nakagami-*m* fading environments. Specifically, exact closed-form formulations of outage probability (OP), ergodic capacity (EC), and average bit error rate (ABER) are derived, supplemented by high signal-to-noise ratio (SNR) asymptotic expansions to yield deeper engineering insights. Monte Carlo simulations verify the exactness of the theoretical analysis and demonstrate the resilience of the proposed HPMA system under realistic keyhole fading scenarios. Our findings reveal that HPMA maintains high performance under severe keyhole conditions, attaining substantial performance superiorities over standard non-orthogonal multiple access (NOMA), particularly regarding outage behavior and error rates. Finally, this study highlights HPMA as a promising, low-complexity, and resource-efficient radio access technology for next-generation wireless communications subjected to keyhole propagation constraints.

## 1 Introduction

The prompt expansion of Internet of Things (IoT) scenarios, including smart industrial manufacturing, automated transportation, environmental monitoring, and digital healthcare, has driven a massive rollout of interconnected devices [1–4]. With this unprecedented device density, combined with heterogeneous traffic characteristics and strict quality-of-service (QoS) requirements, severely intensifies global spectrum scarcity. To solve this challenge, non-orthogonal multiple access (NOMA) has been widely recognized as a pivotal radio access technology designed to fulfill stringent wireless demands, such as ultra-low latency, massive connectivity, high throughput, and robust communication reliability [5–8]. In contrast to traditional orthogonal multiple access (OMA) strategies that assign orthogonal time-frequency resource slots to individual users, NOMA allows multiple users to share identical resource blocks concurrently by exploiting power-domain multiplexing. By leveraging superposition coding at the transmitter alongside successive interference cancellation (SIC) at the receiver side, NOMA substantially boosts spectral efficiency, ensures user fairness, and accommodates massive connectivity, establishing itself as a prominent framework for 5G-and-beyond networks. Nevertheless, NOMA efficiency intrinsically relies on ideal SIC execution, which imposes significant decoding complexity, heightened vulnerability to channel estimation errors, and dangerous error propagation. To tackle the operational drawbacks of SIC processing in NOMA, modern literature has introduced hybrid power-frequency multiple access (HPMA) [9–11], which merges power-domain NOMA concepts with frequency-domain resource allocation, as well as partial NOMA [12,13], which adopts a flexible bandwidth-splitting strategy between OMA and NOMA principles.

Recently, a wide body of literature on NOMA networks has concentrated on deriving exact closed-form expressions for performance metrics, particularly outage probability (OP) and ergodic capacity (EC). To further enhance the performance limits of NOMA, multiple-input multiple-output (MIMO) antenna architectures have been extensively integrated into NOMA designs [14–17]. Specifically, the study in [14] presented a joint maximum-ratio transmission and receive-antenna-selection (MRT/RAS) framework tailored for MIMO-NOMA networks, offering closed-form and high-SNR asymptotic OP evaluations while accounting for residual hardware impairments (RHIs), channel estimation errors (CEEs), and imperfect SIC (ipSIC). Additionally, the authors in [15] investigated transmit power estimation, precoding strategies, and base-station detection for uplink MIMO-NOMA systems, deriving exact power allocation formulas to satisfy target signal-to-interference-plus-noise ratio (SINR) criteria. To optimize spectral utilization while promoting green, energy-efficient communications, the incorporation of simultaneous wireless information and power transfer (SWIPT) into mmWave beamspace MIMO-NOMA systems was examined in [16], where an energy-efficiency maximization problem was solved under strict power-splitting and allocation limits. Addressing physical layer security in MIMO-NOMA networks under eavesdropping threats [17], derived analytical expressions for secrecy outage probability (SOP) while exploiting maximum-ratio-transmission (MRT) and maximum-ratio-combining (MRC) to fortify legitimate channel capacity.

Nonetheless, the spatial performance gains of MIMO fundamentally rest on the hypothesis of rich multipath scattering that guarantees a full-rank channel matrix. In practical propagation environments, this prerequisite is severely invalidated by the occurrence of the keyhole effect, wherein the channel becomes rank-one or rank-deficient despite having completely uncorrelated signal fading at both transceivers [18]. Keyhole propagation occurs when electromagnetic signals are forced through an isolated physical corridor or scattering bottleneck (e.g., narrow tunnels, small apertures, or single dominant obstacles) prior to expanding into scattering environments. A growing number of studies have addressed system performance under keyhole channel degradation [19–25]. For instance [19], derived exact ergodic capacity formulations for uncorrelated Rayleigh keyhole channels. Meanwhile, closed-form expressions for instantaneous SNR distributions and outage capacities over correlated MIMO keyhole links were established in [23]. The channel statistics of double-fading keyhole channels were analyzed in [20] by deriving the PDF of the SNR associated with the single non-zero matrix eigenvalue. In satellite-terrestrial communications [21], proposed DFT-based matrix beamforming strategies to maintain terminal SINR demands under keyhole-degraded satellite links. For signal classification [22], developed a direct modulation classification scheme for lower-order phase-shift keying (PSK) modes over spatially correlated keyhole Rayleigh channels. Furthermore, the combination of MIMO and hybrid automatic repeat request (HARQ) protocols in vehicle-to-vehicle (V2V) networks subject to keyhole fading was investigated in [24], delivering exact OP derivations for Type-I, chase combining (HARQ-CC), and incremental redundancy (HARQ-IR) mechanisms. Most recently [25], explored ultra-reliable secure short-packet transmission under keyhole fading by combining partial NOMA (PNOMA) with finite blocklength communications, deriving exact secure block error rate (SBLER) and BLER metrics to confirm enhanced latency, secrecy, and reliability over traditional NOMA.

To clearly emphasize the technical novelty and advantages of our proposed MIMO-assisted HPMA architecture under keyhole fading, a comprehensive comparison with existing benchmark literature is summarized in Table 1. From an analytical standpoint, this paper addresses a critical gap in current research by presenting exact closed-form expressions for OP, EC, and average bit error rate (ABER), along with high-SNR asymptotic behavior over severe keyhole Nakagami-*m* channel. From a system perspective, we seamlessly blend MIMO multi-antenna benefits with HPMA. Additionally, in contrast to standard NOMA methods that are severely hampered by high decoding complexity and catastrophic error propagation from SIC, the proposed HPMA framework provides a completely SIC-free detection mechanism, achieving exceptional reliability and elevated spectral efficiency unattainable via conventional NOMA.

**Table 1:** Comparative summary of the proposed framework versus existing literature (✓: addressed; ✗: omitted).

Ref.	Multiple Access	MIMO	Keyhole Effect	SIC-Free	OP & EC	ABER	Asymptotic Analysis
[7]	NOMA	✗	✗	✗	✓	✗	✓
[9]	HPMA	✗	✗	✓	✓	✗	✓
[26]	NOMA	✗	✗	✗	✓	✗	✓
[27]	OMA	✓	✓	✗	✓	✗	✓
[28]	Hybrid NOMA	✗	✗	✗	✓	✗	✗
[29]	NOMA	✗	✗	✗	✗	✓	✗
Our work	HPMA	✓	✓	✓	✓	✓	✓

Driven by these remarkable advantages of HPMA, this work investigates MIMO-assisted HPMA communication networks under severe keyhole fading channels. Concretely, the overall cascaded keyhole channel matrix is modeled as the multiplicative product of the multiple-input single-output (MISO) link from the transmitter to the keyhole and the single-input multiple-output (SIMO) link from the keyhole to the destination user. The main contributions of this study are highlighted as follows:

- We establish a rigorous and unified theoretical framework to evaluate the OP, EC, and ABER under generalized Nakagami-*m* fading conditions. Exact closed-form formulas for OP, EC, and ABER are explicitly derived alongside high-SNR asymptotic expansions, explicitly illuminating the system’s achievable diversity orders and coding gains.
- We analyze the delay-limited throughput performance of HPMA under fixed-rate transmission modes using both closed-form formulations and numerical evaluations.
- Extensive Monte Carlo simulations are performed to validate the exactness of the theoretical derivations and to prove that HPMA significantly surpasses traditional NOMA across OP, EC, and ABER metrics, all while avoiding the receiver complexity and error accumulation inherent to SIC processing.

The rest of this paper is structured as follows. Section 2 presents the system framework for the proposed HPMA network, detailing the keyhole fading channel characteristics and signal structure. Section 3 derives exact and asymptotic OP formulations, as well as delay-limited throughput under fixed-rate conditions. Section 4 provides the exact closed-form EC along with its high-SNR asymptotic approximation. Section 5 develops the exact and asymptotic ABER derivations. Section 7 provides comprehensive Monte Carlo simulation results to validate the analytical framework and highlight HPMA performance gains over NOMA. Finally, Section 8 concludes the paper and outlines future research avenues. For convenience, essential notations adopted across the manuscript are listed in Table 2.

**Table 2:** Summary of Key Notations.

Notation	Description
$U_{i}$	The *i*-th user, i∈{1,2}
$N_{S},N_{i}$	Number of antennas at the base station and user $U_{i}$, respectively
$\alpha_{i},\alpha_{12}$	Exclusive bandwidth portion for $U_{i}$ and shared bandwidth fraction
$\delta_{i}$	Power allocation factor for user $U_{i}$
$x_{i},x_{12}$	Information symbol for $U_{i}$ and superimposed signal on the shared subcarrier
$h_{S},h_{i}$	Channel vectors from BS to the keyhole and from keyhole to $U_{i}$
$P_{S}$	Total transmit power at the BS
$\varrho_{S}$	Transmit signal-to-noise ratio
**w**	Precoding vector at the BS
$\gamma_{i}$	Instantaneous signal-to-interference-plus-noise ratio for $U_{i}$
$\beta_{i}$	Noise power normalization factor for $U_{i}$
$m_{S},m_{i}$	Nakagami-*m* fading shape parameters for the corresponding links
$\lambda_{S},\lambda_{i}$	Mean channel gains
${𝒢}_{i}$	Equivalent cascaded keyhole channel gain
${𝒫}_{U_{i}},{ℛ}_{U_{i}}$	Outage probability and ergodic capacity for $U_{i}$
${𝔻}_{i},{𝕋}_{U_{i}}$	Diversity order and average throughput of $U_{i}$
Γ(·),γ(·,·)	Gamma function and lower incomplete Gamma function
$G_{p,q}^{m,n}[\cdot ]$	Meijer’s G-function

## 2 System model

### 2.1 System architecture

We investigate a downlink communication scenario depicted in Fig 1, where a base station (BS) and two users $U_{i}$ are equipped with $N_{S}$ and $N_{i}$ antennas, respectively, with i∈{1,2}, via a keyhole propagation environment. The keyhole phenomenon creates a constrained scattering structure that may allow signal transmission but tends to suppress spatial diversity. Furthermore, the BS has full knowledge of the channel state information (CSI) of two users. To improve spectral efficiency while maintaining implementation simplicity, the HPMA technique is employed. The key idea behind HPMA is to merge the interference-free nature of OMA with the bandwidth-sharing advantage of NOMA into a unified transmission framework that enables SIC-free detection.

**Fig 1:**
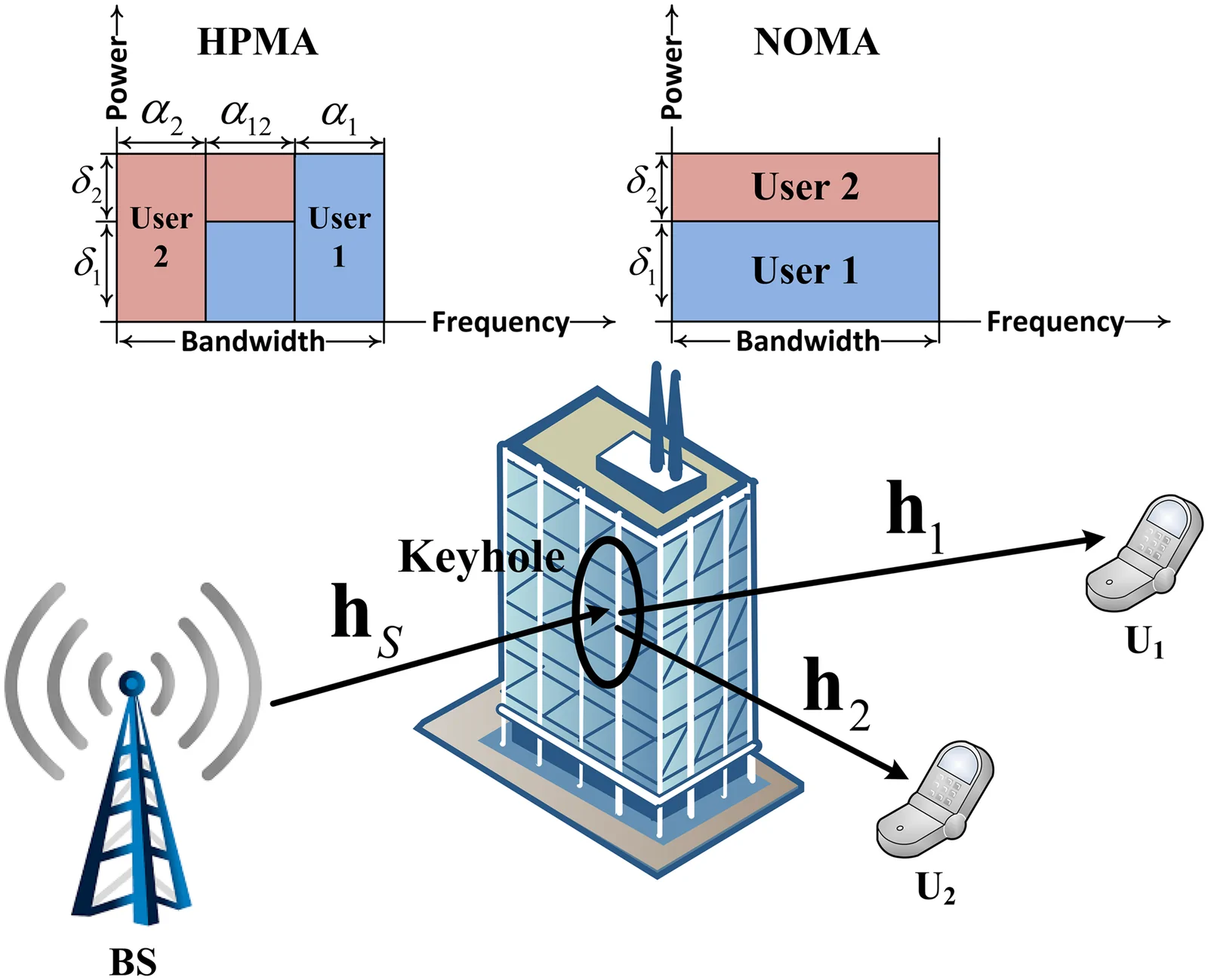
Schematic representation of the proposed HPMA framework over keyhole fading channels.

In the considered system, each user $U_{i}$ is assigned an exclusive subcarrier $s_{i}$, over which a normalized information symbol $x_{i}$ with $𝔼\{|x_{i}{|}^{2}\}=1$ is transmitted across a bandwidth portion $\alpha_{i}$. In addition to these dedicated resources, a shared subcarrier *s*_12_ is introduced for simultaneous transmission of the signal to both users in the bandwidth portion $\alpha_{12}$. The corresponding signal is formed as $x_{12}=\sqrt{\delta_{1}}\;x_{1}+\sqrt{\delta_{2}}\;x_{2},$ where $\delta_{i}$ denotes the power allocation coefficient satisfying $\delta_{1}+\delta_{2}=1$. Furthermore, the bandwidth must satisfy $\alpha_{1}+\alpha_{2}+\alpha_{12}=1$, where the constraint $\alpha_{i}\geq \alpha_{12}$ is imposed to maintain reliable decoding without requiring SIC. If this condition is violated, i.e., $\alpha_{i}<\alpha_{12}$, the interference arising from the shared subcarrier may dominate the desired signal, resulting in a significant degradation of the SINR. In extreme cases, this may lead to an invalid SINR formulation or render reliable decoding infeasible under the considered SIC-free receiver design. Therefore, this constraint is essential to maintain system stability and decoding reliability. At the receiver, the user $U_{i}$ combines the signals from $s_{i}$ and *s*_12_ to detect its desired symbol $x_{i}$, while treating the co-user’s signal $x_{j}$ (j∈{1,2},j≠i) on the shared subcarrier as interference. It is worth noting that when $\alpha_{12}=0$, the proposed scheme reduces to conventional OMA.

To capture the characterization of the wide range of small-scale fading conditions, covering scenarios from severe fading to line-of-sight propagation, we assume that all wireless channels obey independent Nakagami-*m* fading. Due to the structure of the keyhole, the equivalent channel between BS and $U_{i}$ can be modeled as a cascaded product ${\text{h}}_{S}^{†}{\text{h}}_{i}$, where ${\text{h}}_{S}\in {\mathbb{C}}^{N_{S}\times 1}$ denotes the channel matrix from BS to the keyhole and ${\text{h}}_{i}\in {\mathbb{C}}^{N_{i}\times 1}$ denotes the channel matrix from the keyhole to users. This cascaded channel formulation is commonly used to capture the diversity degradation introduced by keyhole propagation. Thus, the received signal of $U_{i}$ at the exclusive subcarrier *s*_1_ and the shared subcarrier *s*_12_ can be given by


$$\begin{array}{c} {\text{y}}_{i}=\left\{ \begin{array}{cc} \sqrt{\alpha_{i}P_{S}}{\text{h}}_{S}^{†}{\text{h}}_{i}\text{w}x_{i}+{\text{n}}_{i}, & {\text{n}}_{i}\sim CN(0,\alpha_{i}\sigma_{i}^{2}{\text{I}}_{N_{S}}),\;\;\;\;s_{i},\\ \sqrt{\alpha_{12}P_{S}}{\text{h}}_{S}^{†}{\text{h}}_{i}\text{w}x_{12}+{\text{n}}_{i}, & {\text{n}}_{i}\sim CN(0,\alpha_{12}\sigma_{i}^{2}{\text{I}}_{N_{S}}),\;\;s_{12}, \end{array}\right. \end{array}$$ (1)

where $P_{S}$ denotes the transmit power at the BS and **w** presents the precoding vector with $\text{w}={\text{h}}_{S}/\|{\text{h}}_{S}\|$. Note that the precoding vector is designed using only the BS-to-keyhole channel. This is justified by the keyhole effect, which renders the MIMO channel effectively rank-one, making the keyhole the dominant propagation bottleneck. As a result, it maximizes the received signal power, while incorporating the keyhole-to-users channel would not yield additional gains but would increase CSI overhead. Based on (1), the instantaneous SINR for detecting $x_{i}$ at the user $U_{i}$ is obtained as


$$\begin{array}{cc} \gamma_{i} & =\frac{(\alpha_{i}+\alpha_{12}\delta_{i})P_{S}\|{\text{h}}_{i}{\|}^{2}\|{\text{h}}_{S}{\|}^{2}}{\alpha_{12}\delta_{j}P_{S}\|{\text{h}}_{i}{\|}^{2}\|{\text{h}}_{S}{\|}^{2}+(\alpha_{i}+\alpha_{12})},\\ & =\frac{(\alpha_{i}+\alpha_{12}\delta_{i})\varrho_{S}\beta_{i}\|{\text{h}}_{i}{\|}^{2}\|{\text{h}}_{S}{\|}^{2}}{\alpha_{12}\delta_{j}\varrho_{S}\beta_{i}\|{\text{h}}_{i}{\|}^{2}\|{\text{h}}_{S}{\|}^{2}+1}, \end{array}$$ (2)

where $\varrho_{S}=P_{S}/\sigma_{i}^{2}$ denotes the transmit SNR, $\delta_{j}=1-\delta_{i}$, and $\beta_{i}=1/(\alpha_{i}+\alpha_{12})$ is introduced to normalize the noise power over the allocated bandwidth. Consequently, the achievable data rate of $U_{i}$ is expressed as


$$\begin{array}{c} {ℛ}_{U_{i}}=\log_{2}\left(1+\gamma_{i}\right). \end{array}$$ (3)

### 2.2 Channel distributions

Since the channel fading amplitudes are modeled via the Nakagami-*m* distribution, the squared channel norm $\|{\text{h}}_{a}{\|}^{2}$ for a∈{S,1,2} follows a Gamma distribution. Accordingly, its probability density function (PDF) and cumulative distribution function (CDF) are expressed, respectively, as


$$\begin{array}{cc} f_{\|{\text{h}}_{a}{\|}^{2}}(x)= & \frac{\Omega_{a}^{N_{a}m_{a}}x^{N_{a}m_{a}-1}}{\Gamma (N_{a}m_{a})}\exp\left(-\Omega_{a}x\right), \end{array}$$ (4)

$$\begin{array}{cc} F_{\|{\text{h}}_{a}{\|}^{2}}(x)= & \frac{\gamma \left(N_{a}m_{a},\Omega_{a}x\right)}{\Gamma (N_{a}m_{a})}. \end{array}$$ (5)

where $\Omega_{a}=m_{a}/\lambda_{a}$, with $m_{a}$ and $\lambda_{a}$ denoting the fading severity parameter and average channel gain, respectively, while Γ(·) and γ(·,·) represent the standard Gamma and lower incomplete Gamma functions, respectively. Following the channel formulation in [30], let $d_{S}$ denote the distance from the BS to the keyhole and $d_{i}$ denote the distance from the keyhole to user $U_{i}$ (i∈{1,2}). The average channel gains are characterized by $\lambda_{S}=d_{S}^{-\zeta }$, $\lambda_{1}=d_{1}^{-\zeta }$, and $\lambda_{2}=d_{2}^{-\zeta }$, where ζ≥2 signifies the path-loss exponent. By defining the cascaded keyhole gain as ${𝒢}_{i}≜\|{\text{h}}_{S}{\|}^{2}\|{\text{h}}_{i}{\|}^{2}$, the associated PDF and CDF can be further formulated in exact closed-form using the Meijer G-function as


$$\begin{array}{cc} f_{{𝒢}_{i}}(x) & =\frac{G_{0,2}^{2,0}\left(x\Omega_{S}\Omega_{i}|\begin{array}{c} -\\ N_{S}m_{S},N_{i}m_{i} \end{array}\right)}{\Gamma (N_{S}m_{S})\Gamma (N_{i}m_{i})x}, \end{array}$$ (6)

$$\begin{array}{cc} F_{{𝒢}_{i}}(x) & =\frac{G_{1,3}^{2,1}\left(x\Omega_{S}\Omega_{i}|\begin{array}{c} 1\\ N_{S}m_{S},N_{i}m_{i},0 \end{array}\right)}{\Gamma (N_{S}m_{S})\Gamma (N_{i}m_{i})}. \end{array}$$ (7)

The above formulation highlights a key feature of keyhole channels. Although each hop may exhibit multipath scattering, the multiplicative structure of the cascaded fading significantly restricts diversity, which generally increases the probability of severe fading events and therefore strongly influences system performance.

## 3 Performance analysis

### 3.1 Outage analysis

Denoting $R_{i}$ as the target transmission rate (bps/Hz) for user $U_{i}$, an outage occurs when the instantaneous achievable rate fails to satisfy this target. Accordingly, the OP associated with $U_{i}$ is formulated as


$$\begin{array}{c} {𝒫}_{U_{i}}=\mathrm{Pr}({ℛ}_{U_{i}}<R_{i})=\mathrm{Pr}(\gamma_{i}<2^{R_{i}}-1≜\gamma_{{th}_{i}}), \end{array}$$ (8)

where $\gamma_{th}$ denotes the SINR threshold corresponding to the required data rate. Substituting the SINR expression in (2) into (8), the OP of the user $U_{i}$ can be rewritten as


$$\begin{array}{cc} {𝒫}_{U_{i}} & =\mathrm{Pr}(\frac{(\alpha_{i}+\alpha_{12}\delta_{i})\varrho_{S}\beta_{i}{𝒢}_{i}}{\alpha_{12}\delta_{j}\varrho_{S}\beta_{i}{𝒢}_{i}+1}<\gamma_{th})=F_{{𝒢}_{i}}(\Phi_{i}), \end{array}$$ (9)

where $\Phi_{i}=\frac{\gamma_{{th}_{i}}}{\left[(\alpha_{i}+\alpha_{12}\delta_{i})-\gamma_{{th}_{i}}\alpha_{12}\delta_{j}\right]\varrho_{S}\beta_{i}}$. For the above formulation to remain valid, the denominator term must satisfy $(\alpha_{i}+\alpha_{12}\delta_{i})>\gamma_{{th}_{i}}\alpha_{12}\delta_{j}$. By utilizing the CDF of ${𝒢}_{i}$ provided in (7), the OP is analytically derived in exact closed-form as


$$\begin{array}{cc} {𝒫}_{U_{i}} & =\frac{G_{1,3}^{2,1}\left(\Phi_{i}\Omega_{S}\Omega_{i}|\begin{array}{c} 1\\ N_{S}m_{S},N_{i}m_{i},0 \end{array}\right)}{\Gamma (N_{S}m_{S})\Gamma (N_{i}m_{i})}. \end{array}$$ (10)

### 3.2 Asymptotic Analysis

When $\varrho_{S}\to \infty$, the asymptotic OP for user $U_{i}$ is derived as [31]


$$\begin{array}{c} {𝒫}_{U_{i}}^{\infty }\approx \frac{\sqrt{\pi }2^{1-2m_{0}}}{\Gamma (N_{S}m_{S})\Gamma (N_{i}m_{i})m_{0}}{\left(4\Phi_{i}\Omega_{S}\Omega_{i}\right)}^{m_{0}}, \end{array}$$ (11)

where $\left|m_{i}-m_{S}\right|=0.5$ and $m_{0}=\min(N_{S}m_{S},N_{i}m_{i})$ represents the diversity order. This expression indicates that the system diversity is dictated by the hop with the minimum number of antennas or fading severity, which is consistent with the physical characteristics of keyhole propagation. From (11), the diversity order ${𝔻}_{i}$ of user $U_{i}$ is defined as


$$\begin{array}{c} {𝔻}_{i}=-\lim_{\varrho_{S}\to \infty }\frac{\log({𝒫}_{U_{i}}^{\infty })}{\log(\varrho_{S})}=m_{0}=\min(N_{S}m_{S},N_{i}m_{i}). \end{array}$$ (12)

**Remark 1.** From (11), the asymptotic OP of user $U_{i}$ holds with the condition $(\alpha_{i}+\alpha_{12}\delta_{i})-\gamma_{{\text{th}}_{i}}\alpha_{12}\delta_{j}>0$. This condition ensures that the desired signal remains dominant over interference, maintaining a valid SINR and enabling reliable decoding. From (12), in keyhole channels, where diversity is limited by $m_{0}=\min(N_{S}m_{S},N_{i}m_{i})$, the system becomes more sensitive to fading. In this case, NOMA suffers from error propagation due to its reliance on SIC. In contrast, HPMA avoids SIC and retains a dedicated transmission component, resulting in more stable and lower OP under severe channel conditions.

### 3.3 Throughput analysis

With the closed-form OP formulations established, we proceed to analyze the throughput behavior of the investigated HPMA network operating in delay-limited mode. Under this operational assumption, each user $U_{i}$ maintains a constant rate $R_{i}$, and reliable signal recovery depends entirely on the instantaneous channel capacity exceeding this target threshold. Thus, the average throughput achieved by user $U_{i}$ corresponds to the target rate scaled by the probability of successful transmission, leading to


$$\begin{array}{c} {𝕋}_{U_{i}}=R_{i}(1-P_{U_{i}}),\;\;\;\;i\in \{1,2\}, \end{array}$$ (13)

where ${𝒫}_{U_{i}}$ denotes the OP derived in (10). The formulation in (13) provides a practical measure of long-term data delivery efficiency when fixed-rate transmission is adopted. It also reflects the inherent tradeoff between reliability and spectral utilization. By substituting the exact OP expressions into (13), the throughput performance can be characterized across different SNR regions. These analytical insights will be employed to compare the proposed HPMA scheme with conventional NOMA in Section 7.

## 4 Ergodic capacity analysis

The EC quantifies the long-term expected data rate achievable over random fading channels with an arbitrarily small error probability. For user $U_{i}$, the EC is formally defined as


$$\begin{array}{c} {ℛ}_{i}=𝔼\left\{\log_{2}(1+{\bar{\gamma }}_{U_{i}})\right\}. \end{array}$$ (14)

Alternatively, the EC for user $U_{i}$ can be written in an equivalent integral formulation as


$$\begin{array}{c} {ℛ}_{U_{i}}=\frac{1}{\ln2}\int_{0}^{\infty }f_{\gamma_{U_{i}}}(x)\ln(1+x)\;dx. \end{array}$$ (15)

By evaluating the integral in (15), the exact closed-form expression for the EC is established in the following proposition.

**Proposition 1.** *The ergodic capacity of user* $U_{i}$ *is derived in exact closed-form as*


$$\begin{array}{c} {ℛ}_{U_{i}}=\frac{G_{2,4}^{4,1}\left(\frac{\chi_{i}}{a_{i}+b_{j}}|\begin{array}{c} 0,1\\ N_{S}m_{S},N_{i}m_{i},0,0 \end{array}\right)}{\ln2\Gamma (N_{S}m_{S})\Gamma (N_{i}m_{i})}-\frac{G_{2,4}^{4,1}\left(\frac{\chi_{i}}{b_{j}}|\begin{array}{c} 0,1\\ N_{S}m_{S},N_{i}m_{i},0,0 \end{array}\right)}{\ln2\Gamma (N_{S}m_{S})\Gamma (N_{i}m_{i})}, \end{array}$$ (16)

*where* $a_{i}=\alpha_{i}+\alpha_{12}\delta_{i}$, $b_{j}=\alpha_{12}\delta_{j}$*, and* $\chi_{i}=\Omega_{0}\Omega_{i}/[\varrho_{S}\beta_{i}]$.

*Proof.* To evaluate (15), we first determine the PDF of the instantaneous SINR $\gamma_{i}$, which is derived by taking the derivative of its corresponding CDF, yielding


$$\begin{array}{cc} f_{\gamma_{i}}(x) & =\frac{\partial }{\partial x}F_{\gamma_{i}}(x)\\ & =\frac{a_{i}}{\varrho_{S}\beta_{i}(a_{i}-b_{j}x{)}^{2}}f_{{𝒢}_{i}}\left(\frac{a_{i}x}{\varrho_{S}\beta_{i}(a_{i}-b_{j}x)}\right), \end{array}$$ (17)

which is valid for $x<\frac{a_{i}}{b_{j}}$. By substituting the cascaded channel PDF $f_{{𝒢}_{i}}(\cdot )$ from (6) into (17), the explicit PDF of $\gamma_{i}$ becomes


$$\begin{array}{cc} f_{\gamma_{i}}(x)= & \frac{a_{i}}{\Gamma (N_{S}m_{S})\Gamma (N_{i}m_{i})x(a_{i}-b_{j}x)}\\ & \times G_{0,2}^{2,0}\left(\frac{\chi_{i}x}{a_{i}-b_{j}x}|\begin{array}{c} -\\ N_{S}m_{S},N_{i}m_{i} \end{array}\right), \end{array}$$ (18)

Substituting (18) into (15), the EC expression is calculated as


$$\begin{array}{cc} {ℛ}_{U_{i}}= & \frac{a_{i}}{\ln2\Gamma (N_{S}m_{S})\Gamma (N_{i}m_{i})}\int_{0}^{a_{i}/b_{j}}\frac{\ln(1+x)}{x(a_{i}-b_{j}x)}\\ & \times G_{0,2}^{2,0}\left(\frac{\chi_{i}x}{a_{i}-b_{j}x}|\begin{array}{c} -\\ N_{S}m_{S},N_{i}m_{i} \end{array}\right)dx. \end{array}$$ (19)

By applying the transformation $t=\frac{x}{a_{i}-b_{j}x}$ and expressing the logarithmic term using its Meijer-*G* representation, i.e., $\ln(1+x)=G_{2,2}^{1,2}\;\left(x|\begin{array}{c} 1,1\\ 1,0 \end{array}\right)$, the integral in (19) can be reformulated as


$$\begin{array}{cc} {ℛ}_{U_{i}}= & \int_{0}^{\infty }\frac{G_{2,2}^{1,2}\left((a_{i}+b_{j})t|\begin{array}{c} 1,1\\ 1,0 \end{array}\right)G_{0,2}^{2,0}\left(\chi_{i}t|\begin{array}{c} -\\ N_{S}m_{S},N_{i}m_{i} \end{array}\right)}{\ln2\Gamma (N_{S}m_{S})\Gamma (N_{i}m_{i})t}dt\\ & -\int_{0}^{\infty }\frac{G_{2,2}^{1,2}\left(b_{j}t|\begin{array}{c} 1,1\\ 1,0 \end{array}\right)G_{0,2}^{2,0}\left(\chi_{i}t|\begin{array}{c} -\\ N_{S}m_{S},N_{i}m_{i} \end{array}\right)}{\ln2\Gamma (N_{S}m_{S})\Gamma \ln2\Gamma (N_{S}m_{S})\Gamma (N_{i}m_{i})t}dt. \end{array}$$

Applying the integral identities of the Meijer-*G* functions [32], the exact closed-form expression for the EC is derived as in (16), which completes the proof. □

The total EC of the investigated system is calculated by aggregating the individual user capacities, i.e.,


$$\begin{array}{c} {ℛ}_{ove}={ℛ}_{U_{1}}+{ℛ}_{U_{2}}. \end{array}$$ (20)

*High-SNR Region:* When $\varrho_{S}\to \infty$, the SINR converges to a deterministic limit, which can be approximated as $\gamma_{U_{i}}^{\infty }\approx \frac{\alpha_{i}+\alpha_{12}\delta_{i}}{\alpha_{12}\delta_{j}}.$ Hence, the asymptotic EC of each user can be approximated as


$$\begin{array}{cc} {ℛ}_{1}^{\infty } & \approx \log_{2}\left(1+\frac{\alpha_{1}+\alpha_{12}\delta_{1}}{\alpha_{12}\delta_{2}}\right), \end{array}$$ (21a)

$$\begin{array}{cc} {ℛ}_{2}^{\infty } & \approx \log_{2}\left(1+\frac{\alpha_{2}+\alpha_{12}\delta_{2}}{\alpha_{12}\delta_{1}}\right). \end{array}$$ (21b)

Therefore, the overall asymptotic EC can be expressed as


$$\begin{array}{c} {\bar{ℛ}}_{ove}^{\infty }={ℛ}_{U_{1}}^{\infty }+{ℛ}_{U_{2}}^{\infty }. \end{array}$$ (22)

## 5 Average bit error rate analysis

As a fundamental performance metric, the ABER evaluates the transmission reliability of wireless communication systems. For the proposed HPMA architecture, the ABER of user $U_{i}$ can be formulated in terms of the CDF of the instantaneous SINR, denoted by $F_{\gamma_{U_{i}}}(x)$, as follows [31]


$$\begin{array}{c} B_{U_{i}}=\frac{p\sqrt{q}}{2\sqrt{\pi }}\int_{0}^{\infty }\frac{e^{-qx}}{\sqrt{x}}\;F_{\gamma_{U_{i}}}(x)\;dx, \end{array}$$ (23)

where the parameters *p* and *q* are modulation-dependent constants. Specifically, binary phase-shift keying (BPSK) corresponds to *p* = 1 and *q* = 1. For *M*-ary pulse amplitude modulation (PAM), these parameters are given by $p=\frac{2(M-1)}{M}$ and $q=\frac{3}{M^{2}-1}$. In addition, for *M*-ary phase-shift keying (PSK), they are specified as *p* = 2 and $q=\sin^{2}\;\left(\frac{\pi }{M}\right)$ [31]. The analytical evaluation of the ABER for user $U_{i}$ is established in the following proposition.

**Proposition 2.** *The exact closed-form expression for the ABER of user* $U_{i}$ *in the investigated HPMA system operating over keyhole Nakagami-m fading channels is derived as*


$$\begin{array}{cc} B_{U_{i}}= & \frac{p}{2\sqrt{\pi }\Gamma (N_{S}m_{S})\Gamma (N_{i}m_{i})}\sum_{r=0}^{\infty }\frac{(-1{)}^{r}}{r!\Gamma (r+1.5)}{\left(\frac{qa_{i}}{b_{j}}\right)}^{r+0.5}\\ & \times G_{2,4}^{3,2}\left(\frac{\chi_{i}}{b_{j}}|\begin{array}{c} 0.5-r,1\\ 1,N_{S}m_{S},N_{i}m_{i},0 \end{array}\right)+\frac{p}{2\sqrt{\pi }}\Gamma \;\left(0.5,\frac{qa_{i}}{b_{j}}\right), \end{array}$$ (24)

*where* $a_{i}$ *and* $b_{j}$ *are defined below (16).*

*Proof.* Substituting the CDF expression in (10) into (23), the ABER of $U_{i}$ can be rewritten as


$$\begin{array}{cc} B_{U_{i}} & =\frac{p\sqrt{q}}{2\sqrt{\pi }\Gamma (N_{S}m_{S})\Gamma (N_{i}m_{i})}\int_{0}^{\frac{a_{i}}{b_{j}}}\frac{e^{-qx}}{\sqrt{x}}\\ & \;\times G_{1,3}^{2,1}\left(\frac{\Omega_{0}\Omega_{i}x}{\varrho_{S}\beta_{i}(a_{i}-b_{j}x)}|\begin{array}{c} 1\\ N_{S}m_{S},N_{i}m_{i},0 \end{array}\right)dx+\frac{p\sqrt{q}}{2\sqrt{\pi }}\int_{\frac{a_{i}}{b_{j}}}^{\infty }\frac{e^{-qx}}{\sqrt{x}}dx. \end{array}$$ (25)

Denote the first integral term in (25) as $I_{1}$. By introducing the transformation $z=\frac{x}{\varrho_{S}\beta_{i}(a_{i}-b_{j}x)}$, $I_{1}$ becomes


$$\begin{array}{cc} I_{1} & =\frac{p\sqrt{q}\sqrt{\varrho_{S}\beta_{i}a_{i}}}{2\sqrt{\pi }\Gamma (N_{S}m_{S})\Gamma (N_{i}m_{i})}\int_{0}^{\infty }\frac{z^{-0.5}}{{\left(1+\varrho_{S}\beta_{i}b_{j}z\right)}^{1.5}}\\ & \;\times e^{-q\frac{\varrho_{S}\beta_{i}a_{i}z}{1+\varrho_{S}\beta_{i}b_{j}z}}G_{1,3}^{2,1}\;\left(\Omega_{0}\Omega_{i}z|\begin{array}{c} 1\\ N_{S}m_{S},N_{i}m_{i},0 \end{array}\right)dz. \end{array}$$ (26)

Next, applying the Taylor expansion $e^{-x}=\sum_{r=0}^{\infty }\frac{(-x{)}^{r}}{r!}$ [33, Eq. (1.211.1)] together with standard integral identities [34, Eq. (07.34.21.0086.01)], $I_{1}$ reduces to


$$\begin{array}{cc} I_{1} & =\frac{p\sqrt{q}}{2\sqrt{\pi }\Gamma (N_{S}m_{S})\Gamma (N_{i}m_{i})}\sum_{r=0}^{\infty }\frac{q^{r}(-1{)}^{r}}{r!\Gamma (r+1.5)}\\ & \;\times {\left(\frac{a_{i}}{b_{j}}\right)}^{r+0.5}G_{2,4}^{3,2}\;\left(\frac{\chi_{i}}{b_{j}}|\begin{array}{c} 0.5-r,1\\ 1,N_{S}m_{S},N_{i}m_{i},0 \end{array}\right). \end{array}$$ (27)

The second integral term in (25), denoted by $I_{2}$, can be evaluated using [35, Eq. (3.351.2)] as


$$\begin{array}{c} I_{2}=\frac{p\sqrt{q}}{2\sqrt{\pi }}\int_{\frac{a_{i}}{b_{j}}}^{\infty }\frac{e^{-qx}}{\sqrt{x}}dx=\frac{p}{2\sqrt{\pi }}\Gamma \;\left(0.5,\frac{qa_{i}}{b_{j}}\right). \end{array}$$ (28)

Finally, substituting (27) and (28) into (25) yields the closed-form ABER expression in (24). □

Though the ABER expression is represented as an infinite series, it exhibits rapid convergence. To verify this property, consider the *r*-th term in (24), defined as


$$\begin{array}{cc} O_{U_{i}}^{\left(r\right)} & =\frac{{\left(\frac{qa_{i}}{b_{j}}\right)}^{r+0.5}}{r!\Gamma (r+1.5)}G_{2,4}^{3,2}\;\left(\frac{\chi_{i}}{b_{j}}|\begin{array}{c} 0.5-r,1\\ 1,N_{S}m_{S},N_{i}m_{i},0 \end{array}\right). \end{array}$$ (29)

Hence, the series in (24) can be expressed as $\sum_{r=0}^{\infty }(-1{)}^{r}O_{U_{i}}^{\left(r\right)}$. Applying the ratio test [36], we evaluate the limit of the absolute ratio between consecutive terms:


$$\begin{array}{cc} \lim_{r\to \infty }\left|\frac{O_{U_{i}}^{\left(r+1\right)}}{O_{U_{i}}^{\left(r\right)}}\right| & =\lim_{r\to \infty }\frac{qa_{i}}{b_{j}(r+1)(r+1.5)}\\ & \;\times \frac{G_{2,4}^{3,2}\left(\frac{\chi_{i}}{b_{j}}|\begin{array}{c} -0.5-r,1\\ 1,N_{S}m_{S},N_{i}m_{i},0 \end{array}\right)}{G_{2,4}^{3,2}\left(\frac{\chi_{i}}{b_{j}}|\begin{array}{c} 0.5-r,1\\ 1,N_{S}m_{S},N_{i}m_{i},0 \end{array}\right)}. \end{array}$$ (30)

To rigorously evaluate the ratio of the Meijer-G functions as r→∞, we invoke its Mellin-Barnes contour integral definition. The parameter dependent on *r* introduces the Gamma function Γ(r+1.5+s) in the numerator of the integrand for the (*r* + 1)-th term, compared to Γ(r+0.5+s) for the *r*-th term. Using the fundamental property of the Gamma function, their ratio is (*r* + 0.5 + *s*), which scales as O(r) for large *r*. Therefore, the ratio of the Meijer-G functions in (30) grows asymptotically at most at a linear rate, i.e., O(r).

Since the denominator polynomial derived from the coefficients, (*r* + 1)(*r* + 1.5), grows at a quadratic rate $O(r^{2})$, the overall limit unconditionally converges to zero:


$$\begin{array}{c} \lim_{r\to \infty }\left|\frac{O_{U_{i}}^{\left(r+1\right)}}{O_{U_{i}}^{\left(r\right)}}\right|\approx \lim_{r\to \infty }\frac{qa_{i}}{b_{j}}\frac{O(r)}{O(r^{2})}=0. \end{array}$$ (31)

Therefore, the absolute convergence of the infinite summation in Proposition 2 is mathematically guaranteed by the ratio test. Furthermore, regarding the convergence rate, the presence of the factorial and Gamma function in the denominator, i.e., r!Γ(r+1.5), drives the terms to decay at a super-exponential rate. Consequently, truncating the infinite series at a relatively small number of terms (e.g., 60 terms) is sufficient to achieve high precision with a negligible truncation error, confirming that the derived ABER expression is highly efficient and practically applicable for numerical evaluations.

## 6 Extension schemes

### 6.1 Multiple users scheme

To generalize the proposed framework to a multi-user scenario, we consider a network with an even number of users *K*. These users are partitioned into *G* distinct pairs, where the *g*-th group is defined as ${𝒢}_{g}=\{U_{1},U_{2}\}$ for g=1,2,...,G. The HPMA protocol is applied independently within each user group, with each pair assumed to experience an independent keyhole fading channel.

For the *g*-th group, the transmitted signaling structure mirrors that of the basic two-user scenario. Accordingly, the received signal at user $U_{i}\in {𝒢}_{g}$ is formulated as


$$\begin{array}{c} {\text{y}}_{i}^{\left(g\right)}=\left\{ \begin{array}{cc} \sqrt{\alpha_{i}P_{S}}{\text{h}}_{S,g}^{†}{\text{h}}_{i,g}{\text{w}}_{g}x_{i}+{\text{n}}_{i}, & {\text{n}}_{i}\sim CN(0,\alpha_{i}\sigma_{i}^{2}{\text{I}}_{N_{S}}),\;\;\;\;s_{i},\\ \sqrt{\alpha_{12}P_{S}}{\text{h}}_{S,g}^{†}{\text{h}}_{i,g}{\text{w}}_{g}x_{12}+{\text{n}}_{i}, & {\text{n}}_{i}\sim CN(0,\alpha_{12}\sigma_{i}^{2}{\text{I}}_{N_{S}}),\;\;s_{12}, \end{array}\right. \end{array}$$ (32)

where ${\text{h}}_{S,g}\in {\mathbb{C}}^{N_{S}\times 1}$ denotes the channel vector from the BS to the *g*-th keyhole, ${\text{h}}_{i,g}\in {\mathbb{C}}^{N_{i}\times 1}$ represents the channel vector from the *g*-th keyhole to user $U_{i}$, and ${𝐰}_{g}=\frac{{\text{h}}_{S,g}}{\left\|{\text{h}}_{S,g}\right\|}$ si*g*nifies the transmit beamforming vector.

Similarly, the instantaneous SINR for detecting $x_{i}$ at the user $U_{i}\in {𝒢}_{g}$ is obtained as


$$\begin{array}{c} \gamma_{i}^{\left(g\right)}=\frac{(\alpha_{i}+\alpha_{12}\delta_{i})\varrho_{S}\beta_{i}\|{\text{h}}_{i,g}{\|}^{2}\|{\text{h}}_{S,g}{\|}^{2}}{\alpha_{12}\delta_{j}\varrho_{S}\beta_{i}\|{\text{h}}_{i,g}{\|}^{2}\|{\text{h}}_{S,g}{\|}^{2}+1}, \end{array}$$ (33)

Furthermore, the performance analysis of $U_{i}$ for each group follows the same mathematical structure as the two-user case, enabling straightforward scalability to larger systems.

### 6.2 Imperfect CSI scheme

In practical wireless deployments, obtaining ideal CSI is challenging due to channel estimation errors, feedback latency, and coarse quantization effects. To model these channel imperfections accurately, we adopt the widely utilized linear minimum mean square error (LMMSE) estimation framework. Consequently, the actual channel coefficient vector is decomposed as [37]


$$\begin{array}{c} 𝐡=\hat{𝐡}+𝐞, \end{array}$$ (34)

where $\hat{𝐡}$ signifies the estimated channel vector, and $𝐞\sim CN\left(0,\sigma_{e}^{2}𝐈\right)$ represents the channel estimation error vector with variance $\sigma_{e}^{2}$. Notably, under LMMSE estimation, the error variance is upper-bounded by and scales proportionally with the large-scale path loss of the corresponding propagation link.

Accounting for imperfect CSI (ipCSI), the effective cascaded channel from the source to user *i* can be expanded as


$$\begin{array}{cc} {𝐡}_{S}^{†}{𝐡}_{i} & =({\hat{𝐡}}_{S}+{𝐞}_{S}{)}^{†}({\hat{𝐡}}_{i}+{𝐞}_{i}),\\ & ={\hat{𝐡}}_{S}^{†}{\hat{𝐡}}_{i}+\underset{\Delta_{i}}{\underbrace{{\hat{𝐡}}_{S}^{†}{𝐞}_{i}+{𝐞}_{S}^{†}{\hat{𝐡}}_{i}+{𝐞}_{S}^{†}{𝐞}_{i}}}, \end{array}$$ (35)

where $\Delta_{i}$ captures the aggregate cascaded estimation error term. Because the individual error components are governed by the path loss along their respective links, the combined error $\Delta_{i}$ can be modeled as a zero-mean complex Gaussian random variable with variance $\sigma_{\Delta_{i}}^{2}\approx \sigma_{\delta }^{2}(\lambda_{S}\lambda_{i})$ [38]. Here, $\lambda_{S}$ and $\lambda_{i}$ denote the path-loss components of the source-to-keyhole and keyhole-to-user-*i* links, respectively, whereas $\sigma_{\delta }^{2}\in [0,1)$ defines the normalized ipCSI coefficient quantifying the severity of channel estimation inaccuracies.

Consequently, the signal received at user $U_{i}$ can be formulated as


$$\begin{array}{c} {\text{y}}_{i}=\left\{ \begin{array}{cc} \sqrt{\alpha_{i}P_{S}}{\hat{\text{h}}}_{S}^{†}{\hat{\text{h}}}_{i}\text{w}x_{i}+\sqrt{\alpha_{i}P_{S}}\Delta_{i}\text{w}x_{i}+{\text{n}}_{i}, & {\text{n}}_{i}\sim CN(0,\alpha_{i}\sigma_{i}^{2}{\text{I}}_{N_{S}}),\;\;\;\;s_{i},\\ \sqrt{\alpha_{12}P_{S}}{\hat{\text{h}}}_{S}^{†}{\hat{\text{h}}}_{i}\text{w}x_{12}+\sqrt{\alpha_{12}P_{S}}\Delta_{i}\text{w}x_{12}+{\text{n}}_{i}, & {\text{n}}_{i}\sim CN(0,\alpha_{12}\sigma_{i}^{2}{\text{I}}_{N_{S}}),\;\;s_{12}, \end{array}\right. \end{array}$$ (36)

where $\text{w}={\hat{\text{h}}}_{S}/\|{\hat{\text{h}}}_{S}\|$ presents the precoding vector. By treating the channel estimation error as an additional interference term, the instantaneous SINR at user $U_{i}$ to detect $x_{i}$ can be expressed as


$$\begin{array}{c} \gamma_{i}^{\text{ipCSI}}=\frac{(\alpha_{i}+\alpha_{12}\delta_{i})\varrho_{S}\beta_{i}\|{\hat{\text{h}}}_{i}{\|}^{2}\|{\hat{\text{h}}}_{S}{\|}^{2}}{\alpha_{12}\delta_{j}\varrho_{S}\beta_{i}\|{\hat{\text{h}}}_{i}{\|}^{2}\|{\hat{\text{h}}}_{S}{\|}^{2}+\varrho_{S}\beta_{i}\sigma_{\Delta_{i}}^{2}+1}. \end{array}$$ (37)

Based on this modified SINR expression, the analytical framework developed for the ideal case can still be straightforwardly applied to evaluate the performance metrics under imperfect CSI conditions.

### 6.3 Hardware impairments scheme

In practical deployments, transceiver hardware inherently suffers from various physical non-idealities, including power amplifier non-linearities, phase noise, and in-phase/quadrature (I/Q) imbalance. These non-ideal characteristics induce distortion noise that deteriorates the received signal quality. Consequently, accounting for residual hardware impairments (RHIs), the signal received at user $U_{i}$ can be formulated as


$$\begin{array}{c} {\text{y}}_{i}=\left\{ \begin{array}{cc} \sqrt{\alpha_{i}P_{S}}{\text{h}}_{S}^{†}{\text{h}}_{i}\text{w}(x_{i}+\eta_{t})+\eta_{r}+{\text{n}}_{i}, & {\text{n}}_{i}\sim CN(0,\alpha_{i}\sigma_{i}^{2}{\text{I}}_{N_{S}}),\;\;\;\;s_{i},\\ \sqrt{\alpha_{12}P_{S}}{\text{h}}_{S}^{†}{\text{h}}_{i}\text{w}(x_{12}+\eta_{t})+\eta_{r}+{\text{n}}_{i}, & {\text{n}}_{i}\sim CN(0,\alpha_{12}\sigma_{i}^{2}{\text{I}}_{N_{S}}),\;\;s_{12}, \end{array}\right. \end{array}$$ (38)

where $\eta_{t}\sim CN(0,\kappa_{t}^{2})$ and $\eta_{r}\sim CN(0,\kappa_{r}^{2}P_{S}\|{\text{h}}_{i}{\|}^{2}\|{\text{h}}_{S}{\|}^{2})$ denote the distortion noise introduced by transmitter and receiver hardware impairments, respectively, and $\kappa_{t}$, $\kappa_{r}$ represent the level of impairments.

From (38), the instantaneous SINR at user $U_{i}$ to detect $x_{i}$ can be expressed as


$$\begin{array}{c} \gamma_{i}^{\text{HI}}=\frac{(\alpha_{i}+\alpha_{12}\delta_{i})\varrho_{S}\beta_{i}\|{\text{h}}_{i}{\|}^{2}\|{\text{h}}_{S}{\|}^{2}}{\alpha_{12}\delta_{j}\varrho_{S}\beta_{i}\|{\text{h}}_{i}{\|}^{2}\|{\text{h}}_{S}{\|}^{2}+\rho_{S}\beta_{i}\kappa^{2}\|{\text{h}}_{i}{\|}^{2}\|{\text{h}}_{S}{\|}^{2}+1}, \end{array}$$ (39)

where $\kappa^{2}=\kappa_{t}^{2}+\kappa_{r}^{2}$. It can be observed that the hardware impairments introduce an additional distortion term in the denominator that increases with the transmit power, thereby limiting the achievable SINR. Based on this modified SINR expression, the same analytical framework developed for the ideal case can be applied to evaluate the performance metrics under hardware impairments.

## 7 Numerical results

This section presents numerical results to verify the analytical findings and to illustrate the performance of the proposed HPMA scheme over keyhole Nakagami-*m* fading channels. Unless explicitly specified otherwise, all performance curves are generated using Monte Carlo simulations with 10^6^ independent channel realizations, which is sufficient to ensure stable and statistically reliable outcomes.

The principal simulation settings are summarized in Table 3. For a fair comparison, we set the parameter for NOMA as: $\alpha_{1}=\alpha_{2}=0$, $\alpha_{12}=1$, $\delta_{1}=0.8$, and $\delta_{2}=0.2$. Furthermore, the receiver noise power at user $U_{i}$ is calculated according to the practical noise model in [39], expressed as $\sigma_{U_{i}}^{2}=N_{0}+10\log_{10}(BW)+NF\;[dBm]$, where *N*_0_ denotes the noise power spectral density, BW represents the system bandwidth, and NF corresponds to the receiver noise figure. The adopted parameter configuration reflects a typical small-cell communication scenario in which a base station serves nearby users through a keyhole scattering link. Such a setup captures realistic propagation characteristics often observed in dense urban or indoor environments where signal transmission is constrained by limited scattering paths. The selected values therefore provide a representative benchmark for assessing system performance in terms of OP, throughput, EC and ABER, which will be discussed in the following figures.

**Table 3:** Simulation parameter configuration used for performance evaluation.

System Parameter	Symbol	Assigned Value
Bandwidth allocation ratios	$\left\{\alpha_{1},\alpha_{2},\alpha_{12}\right\}$	{0.45, 0.45, 0.1}
Power allocation factors	$\left\{\delta_{1},\delta_{2}\right\}$	{0.5, 0.5}
Required transmission rates	$\left\{R_{1},R_{2}\right\}$	{1.5, 2.5} bit/s/Hz
Receiver noise figure	NF	10 dB
Operating bandwidth	BW	10 MHz
Propagation loss exponent	ζ	2.5
Noise power density	*N*_0_	−174 dBm/Hz
Distance between BS and keyhole	$d_{S}$	15 m
Distance between keyhole and U_1_	*d*_1_	35 m
Distance between keyhole and U_2_	*d*_2_	25 m

Fig 2 illustrates the OP against the transmit power $P_{S}$ (dBm) under two distinct fading conditions: $m_{1}=m_{2}=1.5$ and $m_{1}=m_{2}=2.5$. It is evident that the Monte Carlo simulation results align tightly with the analytical curves obtained via (10), thereby corroborating the exactness of our mathematical derivations. Moreover, the asymptotic trajectories closely track both analytical and simulated curves in the high-SNR regime, confirming the validity of the asymptotic derivations. As the transmit power scales up, the outage probabilities of both users decrease monotonically with a diversity slope of $\min(N_{S}m_{S},N_{i}m_{i})$, which perfectly matches the theoretical diversity analysis in Section 3.B. Additionally, enhancing the fading severity parameter ($m_{1}=m_{2}$) yields a noticeable improvement in outage performance for both users. This behavior occurs because higher values of $m_{1}=m_{2}$ signify milder channel fading conditions, which in turn enhances the instantaneous received SNR and reinforces system communication reliability. Notably, HPMA demonstrates a substantial outage performance advantage over conventional NOMA, achieving transmit power savings of approximately 2.5 dB for $U_{1}$ and 5 dB for $U_{2}$ at a target OP threshold. These significant gains highlight the enhanced power-domain efficiency offered by the HPMA strategy, proving its effectiveness for ultra-reliable communication in integrated IoT networks.

**Fig 2:**
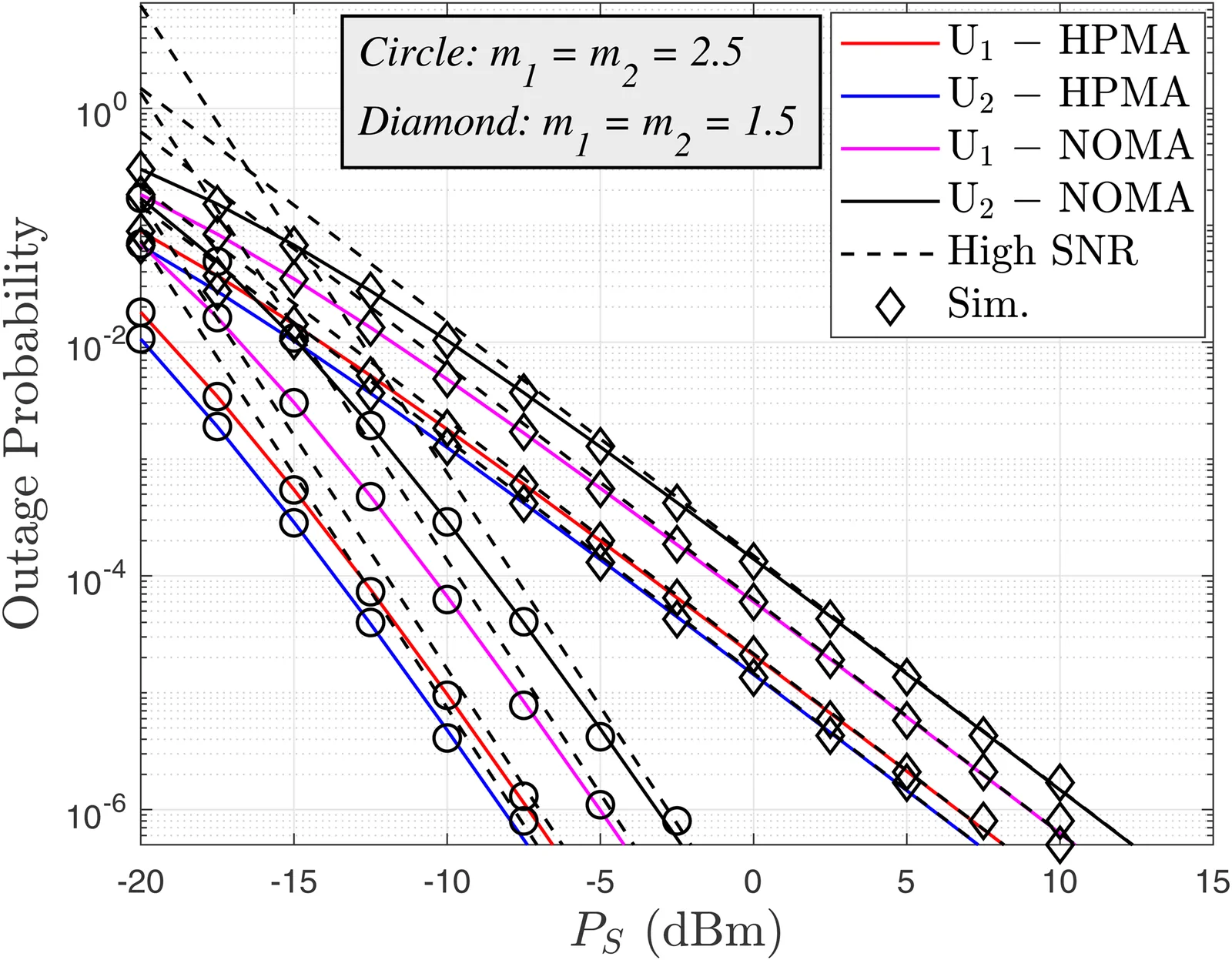
Outage probability versus $P_{S}$ in dBm for $U_{i}$, with $N_{S}=N_{1}=N_{2}=2$.

The impact of the antenna on outage performance is shown in Fig 3. As observed, the OP of both users decreases as the number of antennas increases. This behavior is consistent with expectations, since additional antennas provide higher spatial diversity and enhance the effective channel gain. However, the achievable diversity order remains limited by the presence of the keyhole effect, which restricts the rank of the MIMO channel and consequently reduces the performance benefits that MIMO‑assisted HPMA systems can fully exploit. Fig 4 illustrates the impact of the power allocation coefficient $\delta_{2}$ on the outage performance. As v increases, the OP of $U_{1}$ deteriorates, whereas that of $U_{2}$ improves. This trend is expected because allocating a larger fraction of transmit power to $U_{2}$ directly enhances its received signal strength, resulting in a noticeable reduction in its OP. However, this comes at the expense of $U_{1}$, whose performance degrades due to the reduced available power and increased interference. This result confirms the inherent tradeoff associated with power domain resource allocation in HPMA networks.

**Fig 3:**
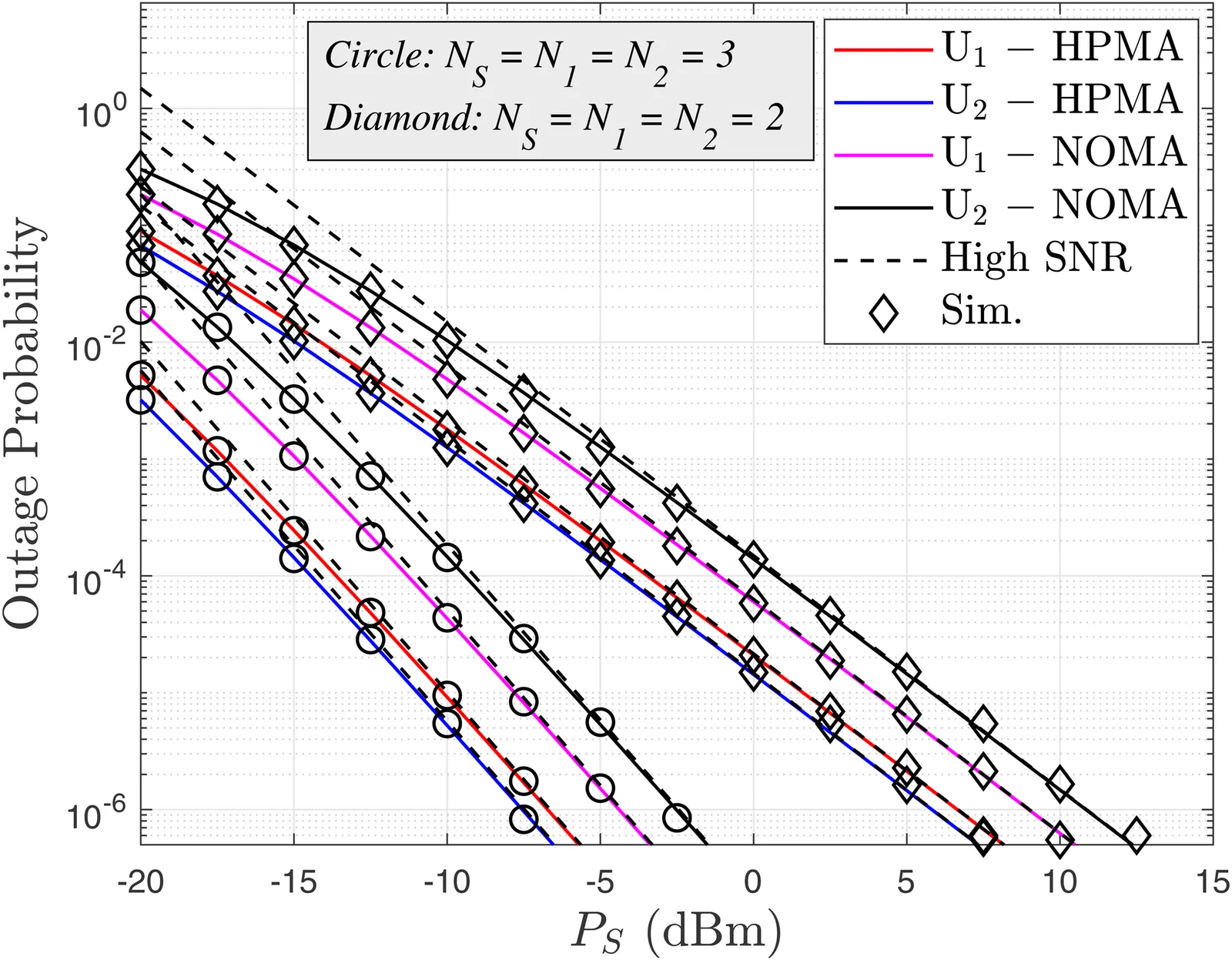
Outage probability versus $P_{S}$ in dBm with $m_{1}=m_{2}=1.5$ and $m_{S}=1$.

**Fig 4:**
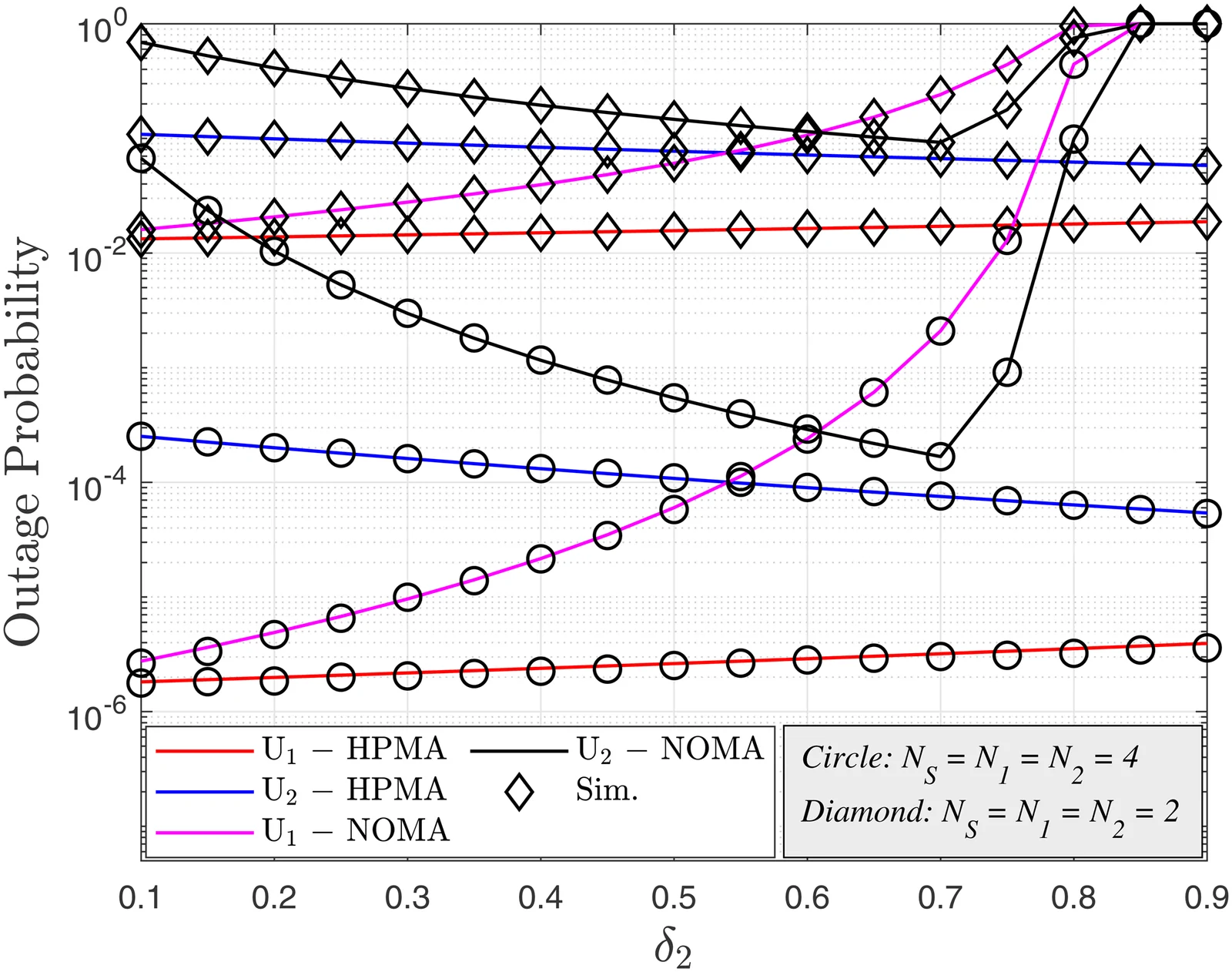
Outage probability versus ($\delta_{2}$) with $m_{1}=m_{2}=1.5$, $m_{S}=1$, *R*_1_ = 0.3, *R*_2_ = 1.2 and $P_{S}=-25$ dBm.

Fig 5 plots the impact of the bandwidth allocation factor $\alpha_{12}$ on the OP of the proposed HPMA system. As can be observed, the results show that the outage performance is strongly influenced by the value of $\alpha_{12}$. Specifically, smaller values of $\alpha_{12}$ (e.g., $\alpha_{12}=0.1$) achieve better outage performance compared to larger values (e.g., $\alpha_{12}=1/3$). This is because increasing $\alpha_{12}$ enlarges the portion of bandwidth allocated to the shared subcarrier, which enhances spectral efficiency but also introduces stronger inter-user interference due to signal superposition. Furthermore, this figure highlights an important design tradeoff: while a larger $\alpha_{12}$ improves bandwidth utilization and supports higher spectral efficiency, it comes at the expense of increased interference and reduced reliability. Therefore, an appropriate choice of $\alpha_{12}$ is essential to balance spectral efficiency and outage performance in HPMA systems.

**Fig 5:**
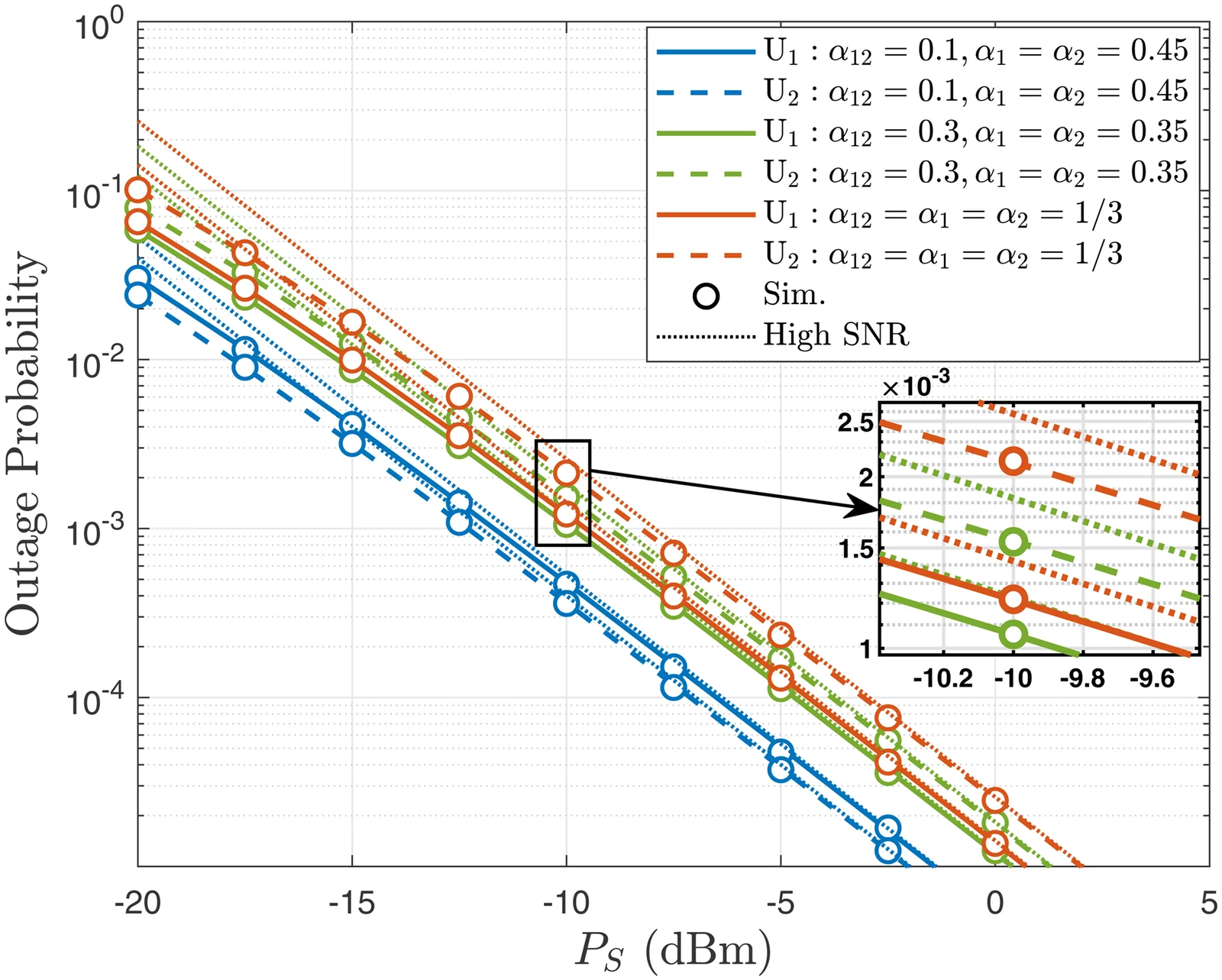
Outage probability versus $P_{S}$ in dBm with $m_{1}=m_{2}=1.5$, $m_{S}=1$, *R*_1_ = 1, *R*_2_ = 1.5 and $N_{S}=N_{1}=N_{2}=2$.

Fig 6 shows the throughput performance of the users versus the target rate $R_{1}=R_{2}$ under different numbers of antennas. It can be observed that throughput of users initially increases as the target rate grows and then begins to decrease once the rate exceeds a certain threshold. This behavior arises because, at low target rates, the system can reliably support the required QoS, leading to a higher effective throughput. However, as the target rate continues to increase, the OP rapidly becomes dominant, causing a significant reduction in the achievable throughput. Consequently, the throughput follows a trend, reflecting the intrinsic tradeoff between rate demands and transmission reliability. In addition, the throughput is further enhanced as the number of antennas increases, owing to the improved spatial diversity and stronger effective channel gains. It is also seen that HPMA consistently achieves higher throughput than NOMA, highlighting the superiority of HPMA in the integrated IoT network.

**Fig 6:**
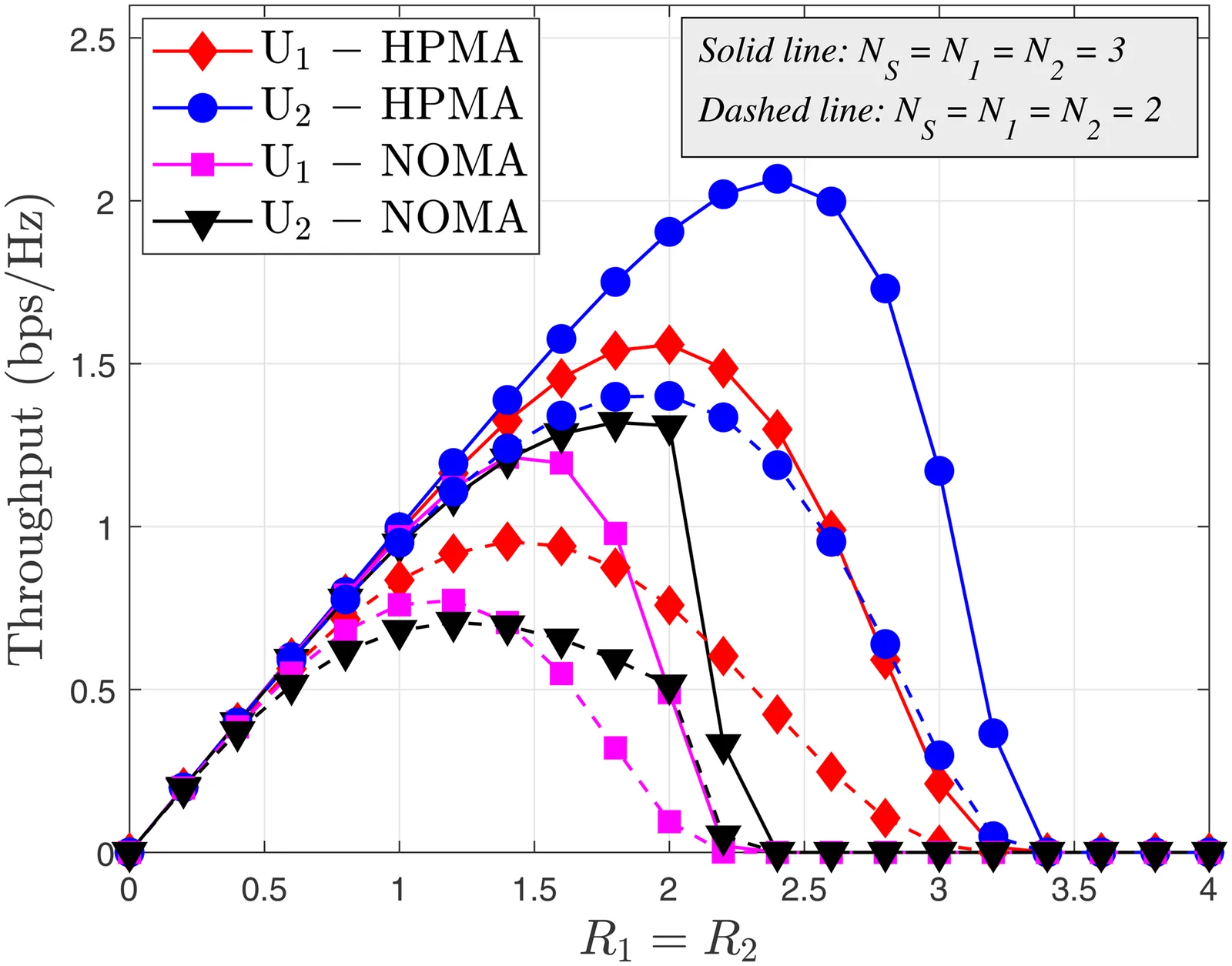
Throughput versus *R*_1_ and *R*_2_, with $m_{S}=1$ and $m_{1}=m_{2}=1.5$.

Fig 7 illustrates the ergodic capacity versus the transmit power $P_{S}$ alongside the benchmark NOMA scheme. It can be observed that the Monte Carlo simulation points align perfectly with the analytical curves across both users, verifying the correctness of the analytical closed-form expressions established in (16). Furthermore, the numerical results demonstrate that the HPMA framework yields a superior ergodic capacity compared to NOMA for user $U_{1}$ over the complete transmit power spectrum, while for user $U_{2}$, HPMA maintains its capacity advantage in the low-SNR region (up to approximately −10 dB). A consistent pattern is evident for the total system ergodic capacity, with HPMA outperforming the NOMA baseline at lower SNR levels (up to roughly 5 dB). These findings underscore the enhanced spectral efficiency provided by HPMA, particularly in power-constrained operating environments.

**Fig 7:**
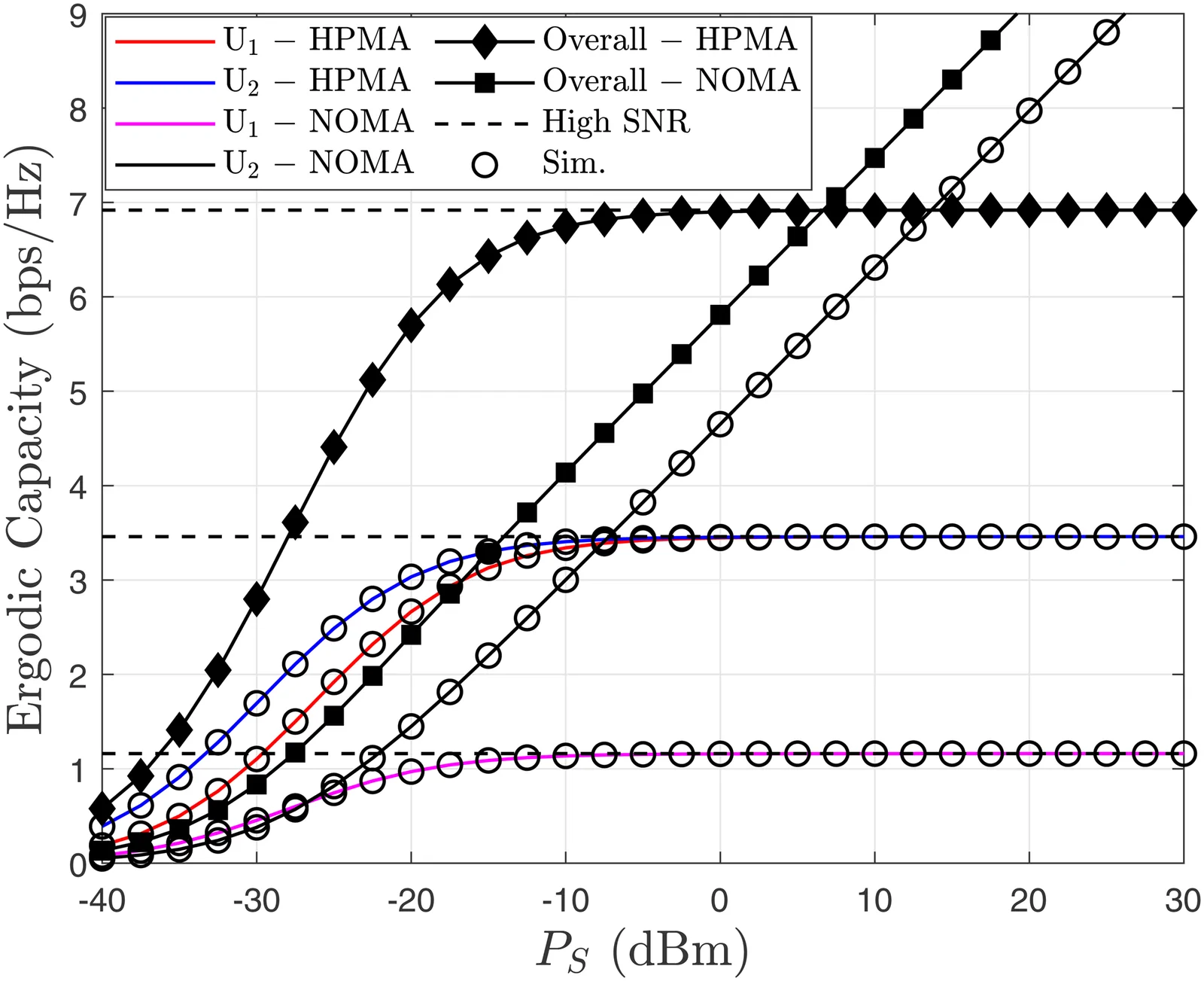
Comparison of ergodic capacity at $U_{i}$ between HPMA and NOMA schemes versus transmit power $P_{S}$ (dBm) with *m*_0_ = 3, $m_{1}=m_{2}=3.5$ and $N_{S}=N_{1}=N_{2}=2$.

Fig 8 depicts the ergodic capacity as a function of the power allocation coefficient $\delta_{2}$. The trend closely resembles that observed in Fig 4, where the ergodic capacity varies nonlinearly with $\delta_{2}$. In particular, the curves reveal the existence of an operating point $\delta_{2}=0.5$ at which both users achieve fairness of the ergodic capacities. This indicates that balances the ergodic capacity of the two users and ensures equitable QoS in HPMA systems.

**Fig 8:**
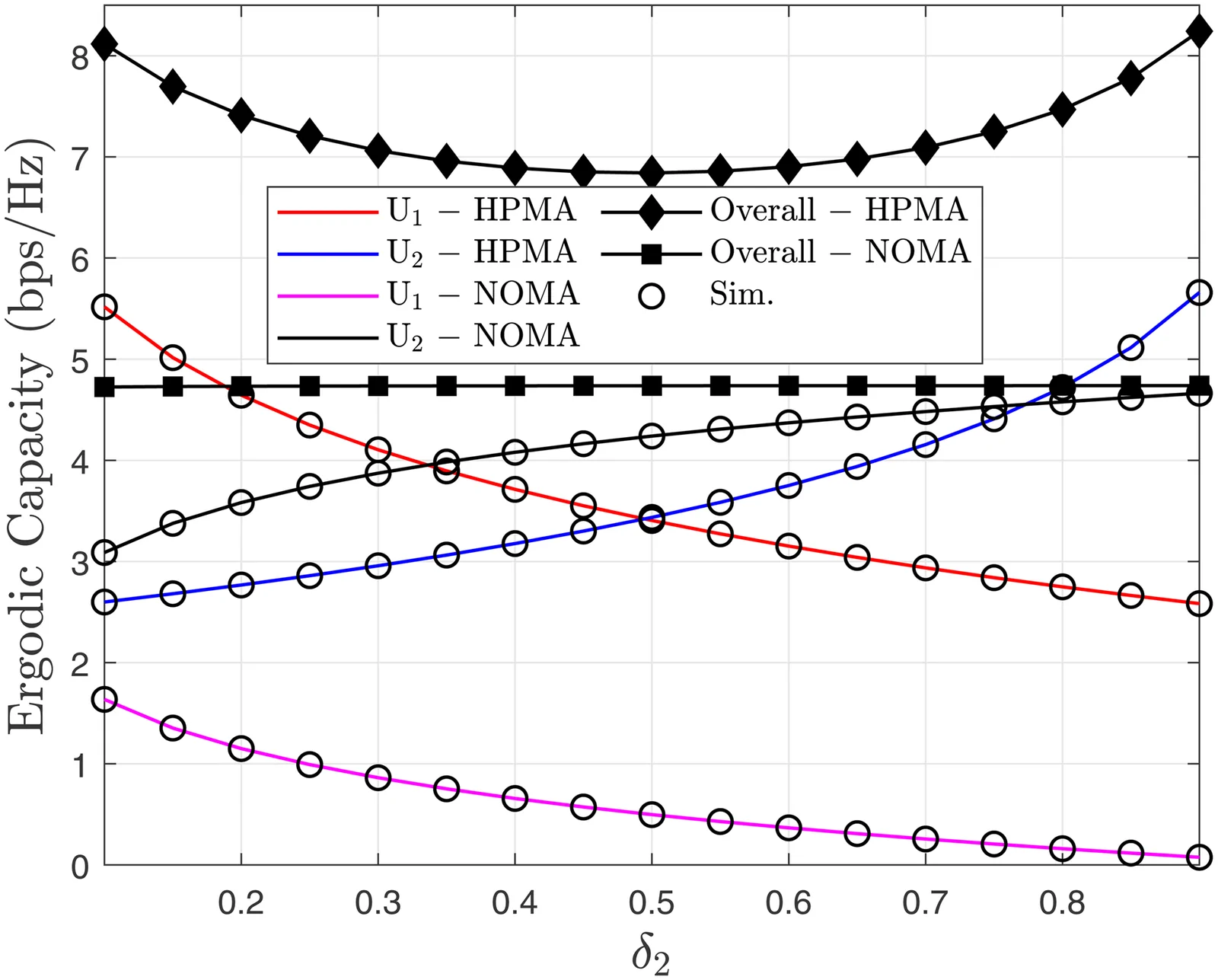
Comparison of ergodic capacity at $U_{i}$ between HPMA and NOMA schemes versus $\delta_{2}$ with, $P_{S}=-10$ dBm, $N_{S}=N_{1}=N_{2}=3$, *m*_0_ = 2 and $m_{1}=m_{2}=2.5$.

Fig 9 plots the ABER performance versus the transmit power $P_{S}$ under two antenna configurations. As can be seen, the ABER of both users decreases and gradually converges as $P_{S}$ and the number of antennas increases, owing to the enhanced signal quality. Moreover, HPMA consistently achieves a lower ABER than NOMA across all considered transmit power levels, further demonstrating its superior reliability. Next, we plot the ABER versus the power allocation $\delta_{2}$ as in Fig 10. The result shows that the average BER (ABER) of *U*_2_ decreases as $\delta_{2}$ increases, whereas the ABER of *U*_1_ exhibits the opposite trend and increases with larger $\delta_{2}$. This behavior reflects the fundamental tradeoff introduced by power allocation in HPMA systems. In addition, HPMA consistently achieves lower ABER than NOMA across the entire range of $\delta_{2}$, demonstrating its superior error‑rate performance.

**Fig 9:**
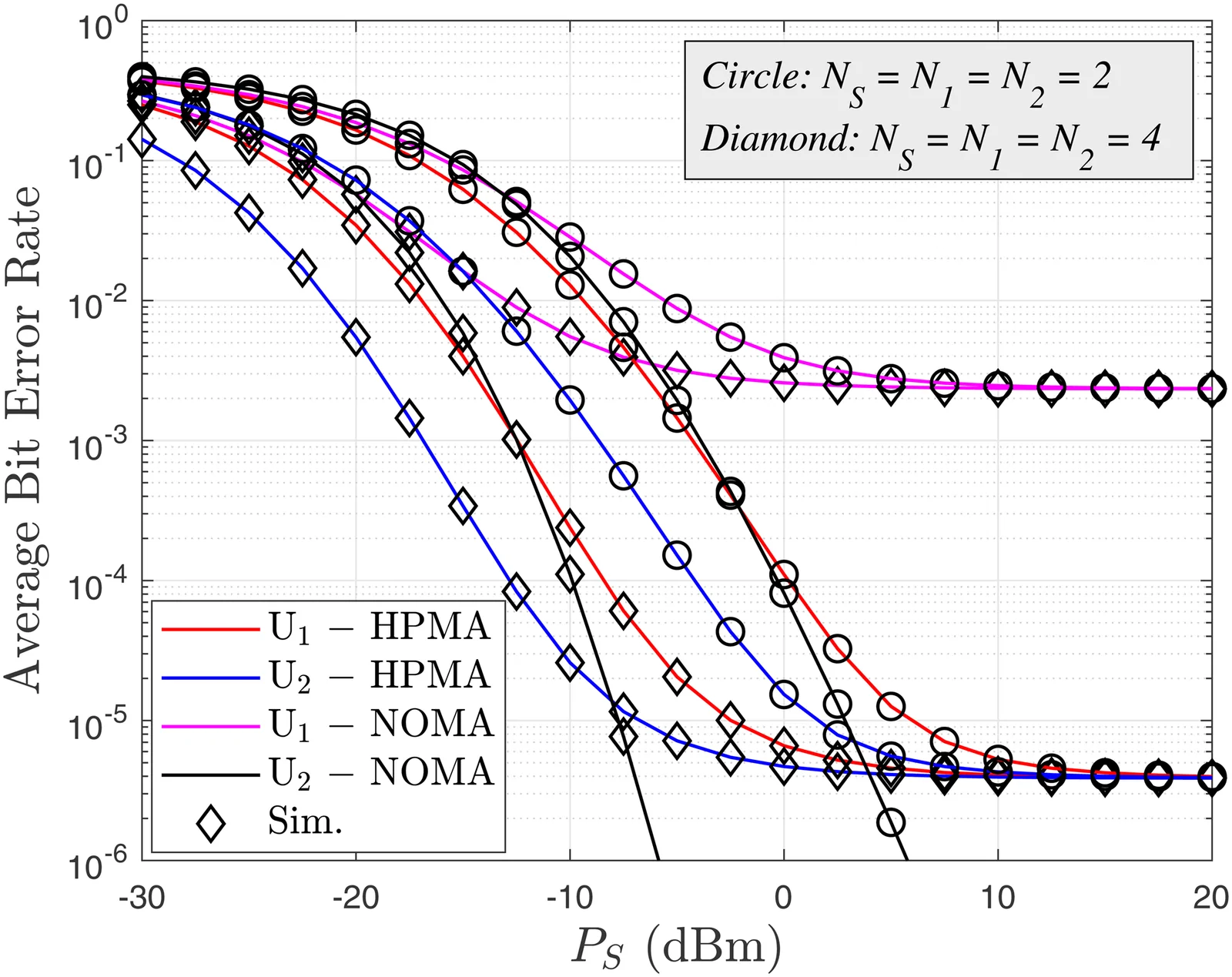
Average bit error rate under BPSK modulation scheme versus $P_{S}$ in dBm, with $m_{S}=2$ and $m_{1}=m_{2}=2.5$.

**Fig 10:**
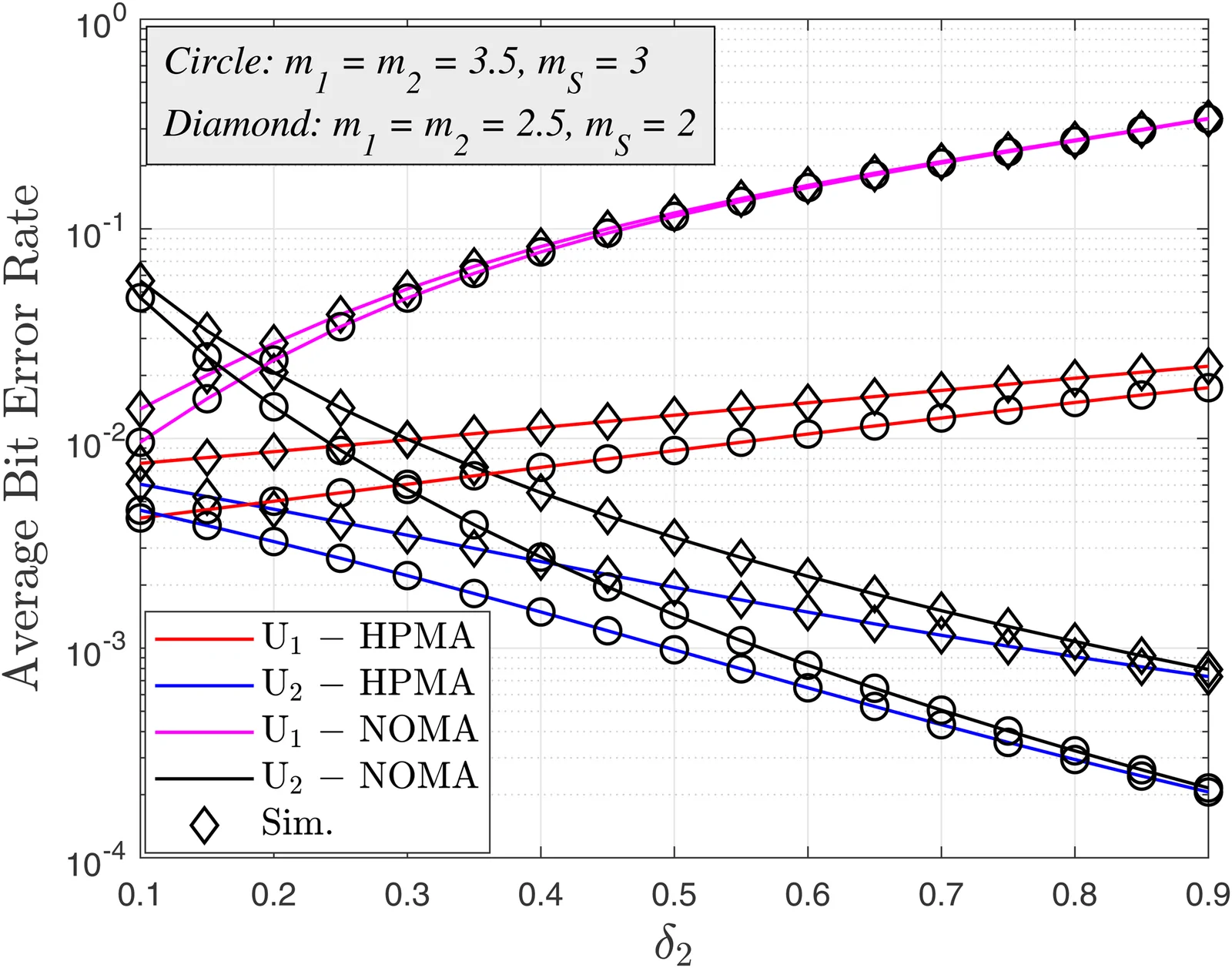
ABER performance under BPSK modulation scheme versus $\delta_{2}$, with $P_{S}=-10$ dBm and $N_{S}=N_{1}=N_{2}=2$.

Fig 11 depicts the impact of the user population *K* on the OP under both HPMA and benchmark NOMA schemes. As observed, the OP for all users degrades markedly with an increasing value of *K*. This outcome is reasonable because scaling up the number of active users intensifies inter-user interference and restricts per-user resource allocation, thereby undermining communication reliability. Moreover, the proposed HPMA framework consistently achieves lower outage probabilities than conventional NOMA for all users throughout the evaluated range of *K*. This superiority becomes particularly prominent in larger *K* regimes, emphasizing the efficacy of HPMA in ultra-dense network deployments. Such performance enhancement stems primarily from the SIC-free detection mechanism of HPMA, which fundamentally eliminates error propagation issues while mitigating receiver decoding complexity compared to NOMA.

**Fig 11:**
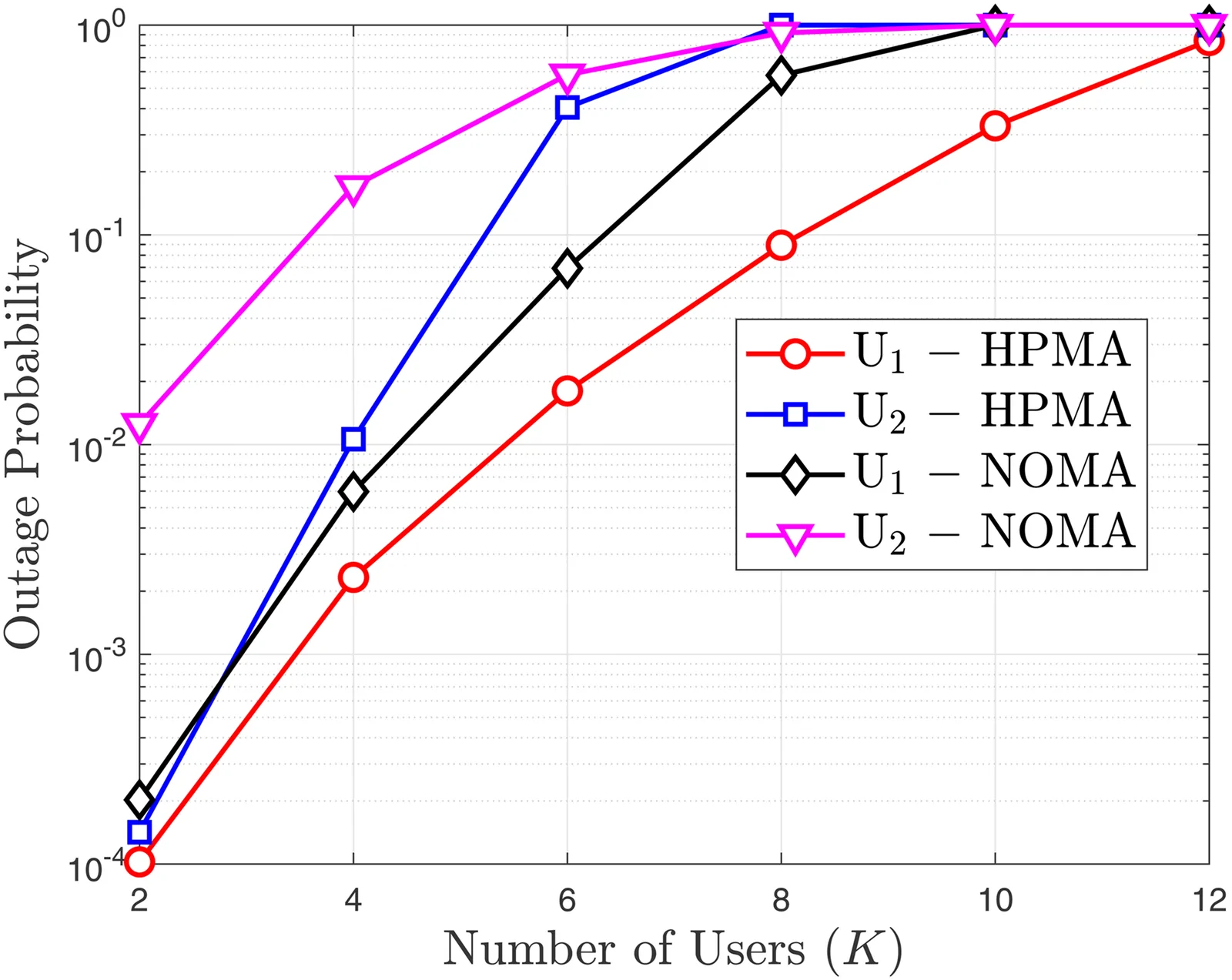
Outage probability versus the number of users *K* for HPMA and NOMA schemes, with $P_{S}=-20$ dBm, $m_{1}=m_{2}=2.5$, $m_{S}=2$, *R*_1_ = 0.5, *R*_2_ = 1, and $N_{S}=N_{1}=N_{2}=2$.

Fig 12 illustrates the influence of imperfect CSI on the OP performance of the proposed HPMA scheme. As observed, under ideal CSI conditions, the OP decreases monotonically with increasing transmit power $P_{S}$, highlighting the crucial role of accurate channel state knowledge in enhancing system reliability. Conversely, in the presence of imperfect CSI, the OP curves experience a pronounced error floor in the high transmit power region. Specifically, across various estimation error variance levels (i.e., $\sigma_{\delta }^{2}=0.01$, 0.05, and 0.1), the OP saturates and ceases to improve despite further increments in $P_{S}$. This saturation effect is driven by the residual interference generated by channel estimation errors, which caps the achievable SINR and becomes the dominant impairment in the high-SNR regime.

**Fig 12:**
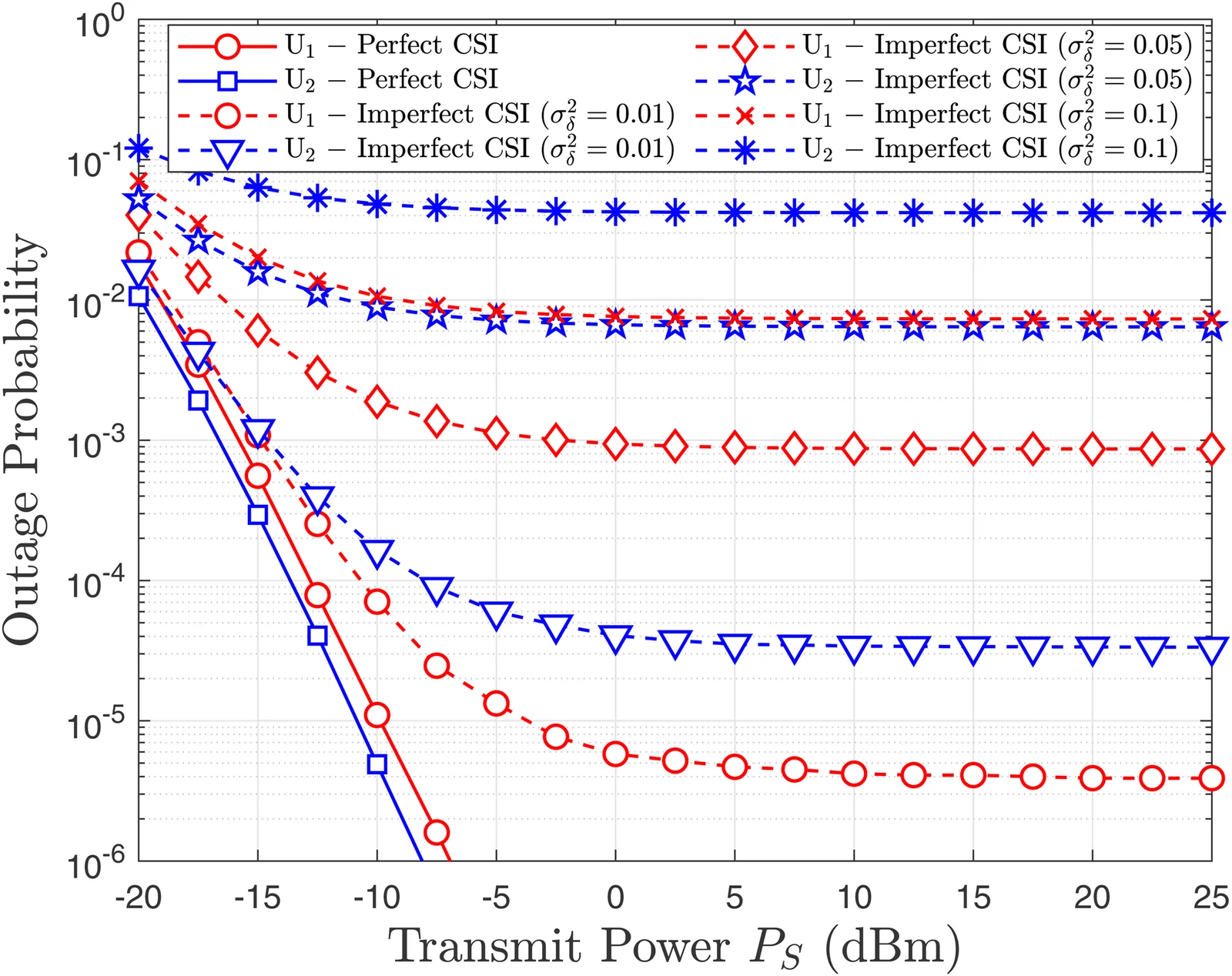
Outage probability versus transmit power $P_{S}$ for the HPMA scheme under perfect CSI and various imperfect CSI error levels ($\sigma_{\delta }^{2}$), with $m_{1}=m_{2}=2.5$, $m_{S}=2$, *R*_1_ = 1.5, *R*_2_ = 2, and $N_{S}=N_{1}=N_{2}=2$.

Lastly, Fig 13 evaluates the impact of RHIs on the OP of the proposed HPMA architecture. The numerical results contrast ideal hardware performance against different impairment severities parameterized by κ. Specifically, as the impairment coefficient grows (e.g., κ=0.1, 0.2, and 0.3), the OP curves shift upward across the entire transmit power range, signifying a notable loss in reliability. This performance degradation originates from distortion noise induced by physical transceiver non-idealities, such as power amplifier nonlinearity, phase noise, and I/Q imbalance. Because these impairments are modeled as additive noise components whose variance scales directly with transmit signal power, they severely constrain the achievable SINR. As a consequence, increasing the transmit power cannot fully offset the reliability degradation caused by hardware non-idealities.

**Fig 13:**
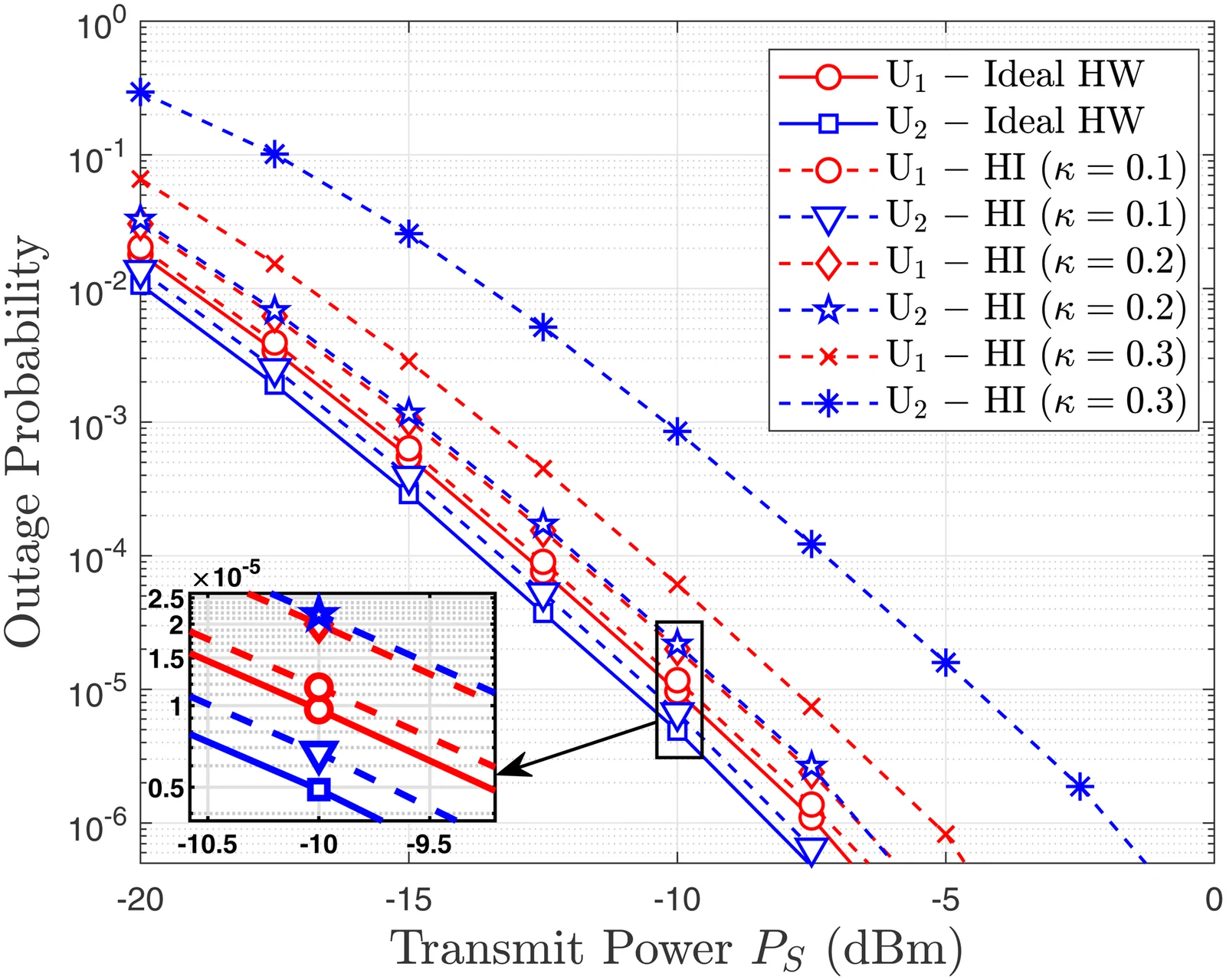
Outage probability versus transmit power $P_{S}$ for the HPMA scheme under ideal hardware and various hardware impairment levels (κ), with $m_{1}=m_{2}=2.5$, $m_{S}=2$, *R*_1_ = 1.5, *R*_2_ = 2, and $N_{S}=N_{1}=N_{2}=2$.

## 8 Conclusion

In this paper, we investigated the performance of MIMO-assisted HPMA networks in the keyhole effect over Nakagami-*m* fading channels. Closed‑form expressions were derived for the OP, EC, and BER, along with their corresponding high-SNR approximations to show the achieved diversity order. In addition, the throughput performance under both delay-limited transmission modes was analytically characterized. The validity of the analytical results was confirmed through extensive Monte Carlo simulations. The results consistently show that the proposed HPMA scheme delivers substantial performance improvements over conventional NOMA, even under severe keyhole conditions that typically degrade MIMO channel rank and system reliability. In particular, HPMA demonstrates enhanced reliability, superior spectral efficiency, and reduced error rates across the main parameters of the proposed system. Furthermore, the findings indicate that HPMA is a robust and efficient multiple‑access solution for future wireless networks deployed in keyhole‑affected environments.
